# Domain dynamics within the PriA DNA helicase regulate DNA replication restart

**DOI:** 10.1093/nar/gkag911

**Published:** 2026-09-22

**Authors:** Haley R Deorio, Alexander T Duckworth, Gavin R Forsythe, Andrew Y Sung, Steve J Sandler, Timothy Grant, James L Keck

**Affiliations:** Department of Biomolecular Chemistry, University of Wisconsin School of Medicine and Public Health, Madison, WI 53711, United States; John and Jeanne Rowe Center for Research in Virology, Morgridge Institute for Research, Madison, WI 53715, United States; Department of Biochemistry, University of Wisconsin-Madison, Madison, WI 53706, United States; Department of Biomolecular Chemistry, University of Wisconsin School of Medicine and Public Health, Madison, WI 53711, United States; Department of Biomolecular Chemistry, University of Wisconsin School of Medicine and Public Health, Madison, WI 53711, United States; Department of Pathology, University of Texas Southwestern Medical Center, Dallas, TX 75390, United States; Department of Microbiology, University of Massachusetts at Amherst, Amherst, MA 01003, United States; John and Jeanne Rowe Center for Research in Virology, Morgridge Institute for Research, Madison, WI 53715, United States; Department of Biochemistry, University of Wisconsin-Madison, Madison, WI 53706, United States; Department of Biomolecular Chemistry, University of Wisconsin School of Medicine and Public Health, Madison, WI 53711, United States

## Abstract

Prematurely terminated DNA replication processes in bacteria must be restarted for successful genome duplication. In *Escherichia coli*, the PriA DNA helicase orchestrates replication restart by assembling the PriA/PriB/DnaT preprimosome complex onto abandoned DNA replication forks and reloading the replicative machinery. We show that the structure of the replication fork lagging strand—whether single- or double-stranded—influences the position of the cysteine-rich region in bound PriA (PriA^CRR^). When PriA binds to synthetic replication forks with a single-stranded lagging strand, the PriA^CRR^ is found either in a state similar to free PriA or in a rotated position that encircles the lagging strand and allows PriB recruitment. Binding to a synthetic replication fork with a duplex lagging strand neither alters the PriA^CRR^ position nor promotes PriB binding, suggesting PriA must unwind duplex lagging-strand DNA to trigger PriA^CRR^ movement and subsequent preprimosome formation. PriA variants designed to destabilize the two PriA^CRR^ positions differentially affect ATPase activity, helicase function, PriB binding *in vitro*, and PriA activity *in vivo*. These results support a regulatory switch model in which the lagging-strand DNA structure modulates the PriA^CRR^ position, thereby governing PriA’s biochemical and cellular activities.

## Graphical Abstract

**Graphical Abstract:**
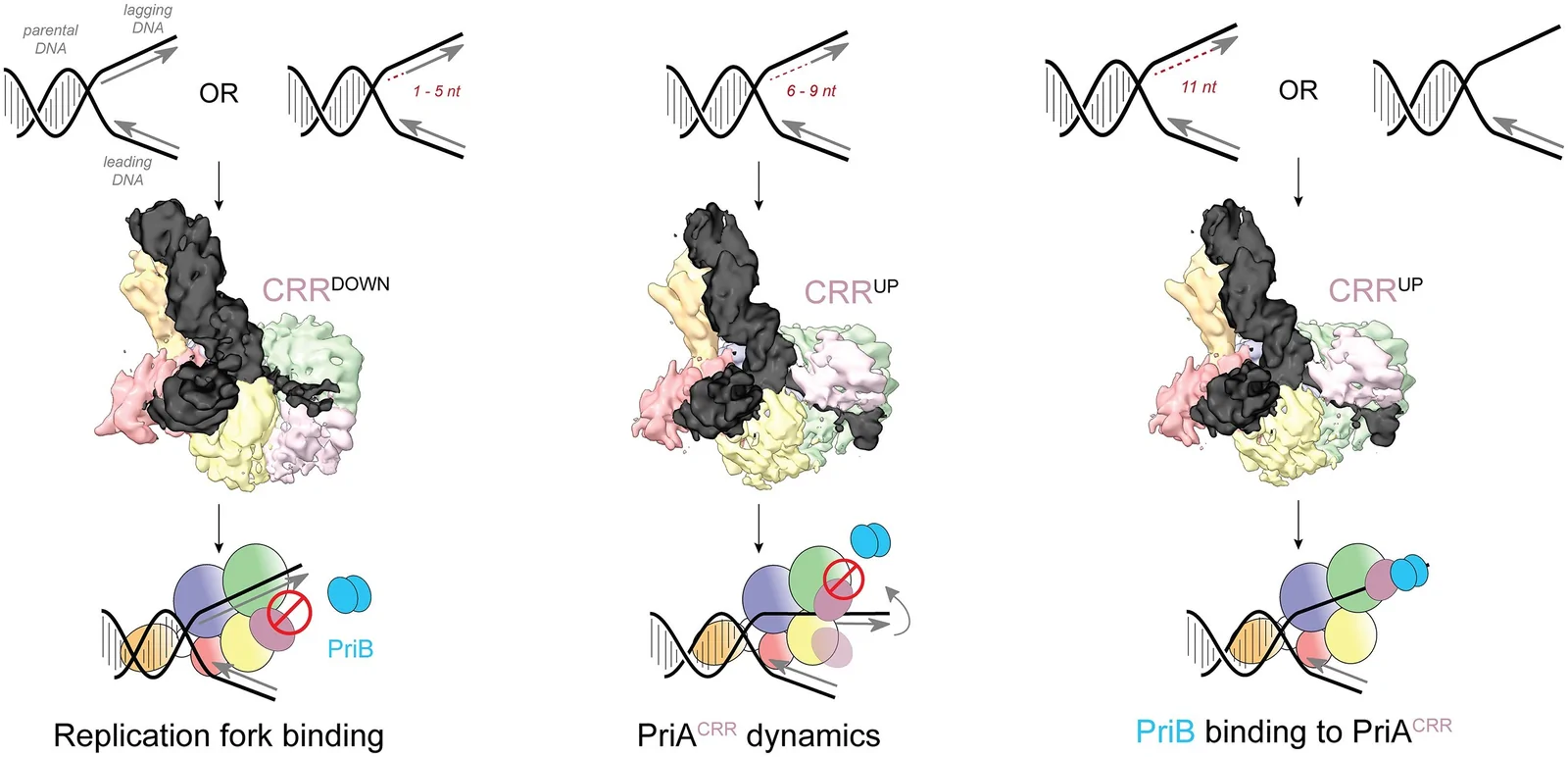

## Introduction

DNA replication is a tightly regulated, high-fidelity process. In *Escherichia coli*, replication begins at the origin of replication (*oriC*), a 250 basepair (bp) sequence within the ∼5 Mbp circular chromosome [1–3]. The DnaA initiator protein recognizes sequence elements within *oriC* and induces origin unwinding, creating a single-stranded DNA (ssDNA) bubble. DnaA then loads the replicative helicase, DnaB, onto each of the ssDNA strands, and DnaB directs assembly of replication complexes (replisomes), launching bidirectional DNA replication [4–6]. Replisomes are frequently challenged by protein roadblocks and damaged DNA that stall replication forks and, in some cases, can lead to replisome disassembly [7–9]. The latter events require replisome reloading onto abandoned replication forks via DNA replication restart pathways.

Reloading of the replication machinery at failed replication sites relies on structure-specific detection of the abandoned replication fork, in contrast to the sequence specificity that restricts replication initiation to *oriC*. While DNA replication initiation at *oriC* is controlled through well-studied mechanisms to ensure fidelity [10], the mechanisms that regulate replisome reloading onto abandoned DNA replication forks are less understood. DNA replication restart pathways must balance the plasticity required to detect diverse replication fork structures with the specificity required to avoid improper initiation events [11, 12].

The PriA/PriB replication restart pathway in *E. coli* is mediated by PriA, PriB, and DnaT, which assemble on replication forks to form a multi-protein complex known as the preprimosome [13–18]. PriA begins replication restart by recognizing the abandoned replication fork structure and remodeling the lagging strand to form ssDNA. Given that strand configurations may differ depending on the event that terminated replication, PriA must recognize and process diverse replication fork structures to ensure DNA replication is completed. On replication forks with ssDNA lagging strands, PriA accomplishes this by binding to and displacing the ssDNA-binding protein (SSB), whereas for double-stranded (ds) DNA lagging-strand structures, PriA relies on its function as a DNA helicase to unwind the duplex [17, 19–23]. SSB can enhance duplex unwinding in the latter case by binding the ssDNA that results from lagging-strand unwinding; this enhancement does not require PriA/SSB interaction when the leading strand is duplex [15, 22, 23]. Physical interaction between PriA and SSB can occur in the presence or absence of DNA [20, 23, 24].

PriB, a homodimeric SSB paralog, is recruited to the PriA/replication fork complex in the next step of preprimosome assembly. Unlike SSB, PriB association with PriA is greatly enhanced by PriA binding to replication forks with ssDNA lagging strands [21, 25–28]. The cysteine-rich region of PriA (PriA^CRR^), a 50-residue insertion within the second helicase domain (PriA^HD2^) that binds two stabilizing zinc ions, mediates PriB binding and facilitates lagging-strand unwinding [16, 21, 29–31]. DnaT binding to the PriA/PriB/DNA complex completes the preprimosome, which is then competent to load DnaB onto the lagging strand to reinitiate DNA replication [25, 31–35]. Alternative pathways that rely on the PriC protein can also function, although genetic studies have demonstrated that PriA/PriB is the primary replication restart pathway in *E. coli* and that it is particularly important in repair of dsDNA breaks [13, 36].

Given that the PriA/PriB/DnaT preprimosome selectively loads DnaB onto abandoned replication fork structures, specific structural features of DNA forks likely serve as determinants of preprimosome assembly and subsequent DnaB loading. PriA binds to each arm of the replication fork and targets its helicase activity to the lagging strand, which is key to this specificity [16, 17, 19, 21, 37, 38]. Replication fork recognition also modulates preprimosome assembly, with DNA binding to PriA strongly enhancing PriB association [30, 31, 39, 40]. These observations suggested that replication fork binding induces a conformational change in PriA that exposes its PriB-binding site [30]. Structures of PriA bound to either PriB or PriB and DnaT together on synthetic DNA replication forks support this model [21, 35]. Within these structures, PriA binding to a replication fork with a ssDNA lagging strand is accompanied by an ∼80° rotation of the PriA^CRR^ and a ∼15° rotation of the second helicase domain (PriA^HD2^) relative to their positions in apo PriA [19–21]. This rearrangement generates a pore in PriA that entraps nine bases of lagging-strand ssDNA and simultaneously exposes the PriB binding site on PriA^CRR^ and the DnaT docking site on PriA^HD2^ and C-terminal domains. Despite these insights, whether the replication fork structure alone influences the position of the PriA^CRR^ in the absence of other preprimosome proteins is not known.

To investigate the mechanisms linking replication fork recognition and preprimosome assembly by PriA, we used cryogenic-electron microscopy (cryo-EM) to examine the structures of *E. coli* PriA bound to replication forks with either ssDNA or dsDNA lagging strands. When bound to a replication fork with a dsDNA lagging strand, the PriA^CRR^ is most often found in a conformation matching the “down” position observed in prior apo PriA structures (CRR^DOWN^) [19, 20]. However, when the lagging strand is ssDNA, the PriA^CRR^ adopts conformations matching either the CRR^DOWN^ position or the rotated CRR^UP^ position observed in the PriA/PriB/replication fork structure [21]. Biochemical studies demonstrate that stable PriB docking onto the PriA^CRR^, which occurs when the domain is in the CRR^UP^ position, requires binding to a replication fork with at least 11 ssDNA lagging-strand bases. *In vitro* and *in vivo* analyses of PriA^CRR^ variants designed to disrupt interdomain contacts that stabilize the two PriA^CRR^ positions reveal differential effects on replication fork binding, DNA unwinding, ATPase activity, and PriB docking. The results collectively define regulatory roles for PriA^CRR^ dynamics that are likely critical for restricting replication restart to replication forks suitable for reinitiation.

## Materials and methods

### Protein purification

Wild-type (WT) *E. coli* PriA containing an N-terminal His-tag and native PriB were expressed and purified as previously described [21, 41].

For PriA^CRR^ variants, BL21(DE3) cells were transformed with an overexpression plasmid and grown in Luria Broth supplemented with 50 µg/mL of ampicillin to an OD_600 nm_ of 0.6 and induced with 1 mM of isopropyl-β-d-1-thiogalactopyranoside for 3 h at 37°C. Cells were pelleted and resuspended in 40 mL of PriA Lysis Buffer (20 mM Tris–HCl, pH 7, 10% glycerol, 500 mM NaCl, 500 mM dextrose, 10 mM imidazole, 1 mM β-mercaptoethanol (BME), 1 mM phenylmethylsulfonyl fluoride (PMSF), 1 mM benzamidine, and 1 ethylenediaminetetraacetic acid (EDTA)-free protease inhibitor tablet). Cells were sonicated and clarified by centrifugation. All purification steps were performed using an Äkta Pure FPLC system (Cytiva) at 4°C. Unfiltered soluble lysate was loaded onto a 5 mL of HisTrap FF crude FPLC column (Cytiva) equilibrated with PriA Wash Buffer (PriA Lysis Buffer excluding protease inhibitors). Non-specific and weakly bound proteins were removed from the column during the application of 50 mL of PriA Wash Buffer. Protein was eluted using 30 mL of PriA Elution Buffer (20 mM Tris–HCl, pH 7, 10% glycerol, 1 M NaCl, 250 mM imidazole, and 1 mM BME). The protein eluate was diluted to 200 mM NaCl with the addition of 45 mL of PriA dilution buffer (10 mM HEPES, pH 7.5, 10% glycerol, and 1 mM BME), and precipitated protein was filtered from soluble protein using 0.22 µm syringe filters (Fisher Scientific). Filtered, soluble protein was directly loaded onto a 20 mL of Hi-Prep Sulfopropyl Sepharose fast flow (SPFF) FPLC column (Cytiva) and eluted using a 100–1000 mM NaCl gradient in SPFF Buffer (10 mM HEPES-HCl, pH 7, 10% glycerol, 100–1000 mM NaCl, and 10 mM dithiothreitol (DTT)). Eluted protein was concentrated and loaded onto a 1 M NaCl SPFF Buffer equilibrated HiPrep S100 FPLC column (Cytiva). Purified protein was concentrated, dialyzed against storage buffer (10 mM HEPES, pH 7, 50% glycerol, 500 mM NaCl, and 10 mM DTT), and stored at −20°C.

*E. coli* PriB containing an N-terminal Maltose Binding Protein (MBP) tag was expressed as described [21, 41]. Frozen cell pellets were resuspended in 40 mL of PriB Lysis Buffer (20 mM Tris–HCl, pH 7, 10% glycerol, 1 M NaCl, 10 mM imidazole, 1 mM BME, 1 mM PMSF, 1 mM benzamidine, and 1 EDTA-free protease inhibitor tablet). Cells were sonicated and clarified by centrifugation. All purification steps were performed using an Äkta Pure FPLC system (Cytiva) at 4°C. Unfiltered soluble lysate was loaded onto a 5 mL of HisTrap FF crude FPLC column (Cytiva) equilibrated with PriB Wash Buffer (PriB Lysis Buffer excluding protease inhibitors). Nonspecific and weakly bound proteins were removed from the column during the application of 50 mL of PriB Lysis Buffer. The protein was eluted from the column using 30 mL of PriB Elution Buffer (20 mM Tris–HCl, pH 7, 10% glycerol, 1 M NaCl, 250 mM imidazole, and 1 mM BME). Eluted protein was applied to a 5 mL of MBPTrap HP FPLC column (Cytiva) at 4°C equilibrated with MBP Binding Buffer (10 mM Tris–HCl, pH 7, 200 mM NaCl, 10% glycerol, 1 mM EDTA, and 1 mM BME), then washed with 50 mL of MBP Binding Buffer to remove any nonspecific or weakly bound proteins. Protein was eluted from the column with 30 mL of MBP Elution Buffer (10 mM Tris–HCl, pH 7, 200 mM NaCl, 10 mM d-maltose, 10% glycerol, 1 mM EDTA, and 1 mM BME). Eluted protein was dialyzed against 3 L of MBP Binding Buffer 2 (10 mM Tris–HCl, pH 7, 10% glycerol, 200 mM NaCl, and 1 mM BME) to remove EDTA and maltose, overnight at 4°C. Dialyzed protein was filtered using 0.22 µm syringe filters (Fisher Scientific), then directly loaded onto a 20 mL of Hi-Prep SPFF FPLC column (Cytiva) equilibrated with SPFF Buffer A (10 mM HEPES–HCl, pH 7, 10% glycerol, 100 mM NaCl, and 10 mM DTT). Protein was eluted using a 100–1000 mM NaCl gradient of SPFF Buffer A and SPFF Buffer B (10 mM HEPES–HCl, pH 7, 10% glycerol, 1 M NaCl, and 10 mM DTT). Eluted protein was concentrated and loaded onto an SPFF Buffer B equilibrated HiPrep S100 FPLC column (Cytiva). Purified protein was concentrated and dialyzed against storage buffer (10 mM HEPES–HCl, pH 7, 50% glycerol, 500 mM NaCl, and 10 mM DTT), and stored at −20°C.

### Cryo-EM sample preparation and imaging

10 nmol PriA and 10 nmol of either Fork 1 (ssDNA lagging strand, Supplementary Table S1) or Fork 2 (dsDNA lagging strand) were co-incubated in 5 mL of S200 Buffer (50 mM Tris–HCl, pH 8, 2 mM DTT, 5 mM EDTA, and 75 mM NaCl) on ice and concentrated to 500 μl. The complex was purified on a Superdex 200 Increase 10/300 GL analytical size exclusion FPLC column (Cytiva) in S200 Buffer. Peak fractions were combined and concentrated to ∼1 mg/mL. Samples with Fork 1 or Fork 2 were applied to AuFlat 1.2/1.3 (Electron Microscopy Sciences) or Ultrafoil 1.2/1.3 (Quantifoil) grids, respectively, that were glow-discharged for 20 s using a GloQube Plus glow discharge system (Qurom Inc). The grids were plunge frozen at 4°C using a Vitrobot Mark VI (ThermoFisher) at 100% humidity, with a blot time of 5.5 s. Movies were collected at 300 kV on a Titan Krios controlled by SerialEM. 1779 movies of the Fork 1 sample and 4221 movies of the Fork 2 sample were collected using a Gatan K3 camera operating in CDS mode and BioQuantum energy filter with a 20 eV slit width at a calibrated pixel size of 0.834 Å/pix. Datasets were collected with a total dose of 65 e/Å^2^ split into 65 fractions. Images were collected with a defocus range of 0.5–2.5 μm.

### Data processing and EM density map generation

Movies were imported into cisTEM [42] 2.065 for data processing following the standard cisTEM processing workflow (Supplementary Fig. S1A). After motion correction and contrast transfer function (CTF) parameter estimation, only micrographs with a detected CTF fit resolution better than 3.5 Å (Fork 1 data) or 4.0 Å (Fork 2 data) were kept for further processing. This left 1, 228 Fork 1 movies and 3, 289 Fork 2 movies for further processing. Initially, ∼800 000 and ∼1 200 000 particles were picked from the Fork 1 and Fork 2 datasets, respectively, using the cisTEM disc picker and subjected to 2D classification. While the Fork 1 dataset was 2D classified *ab initio*, the Fork 2 dataset was classified using seeds projected from an earlier 200 kV Talos Arctica volume (Supplementary Fig. S1B).

**Figure 1.**
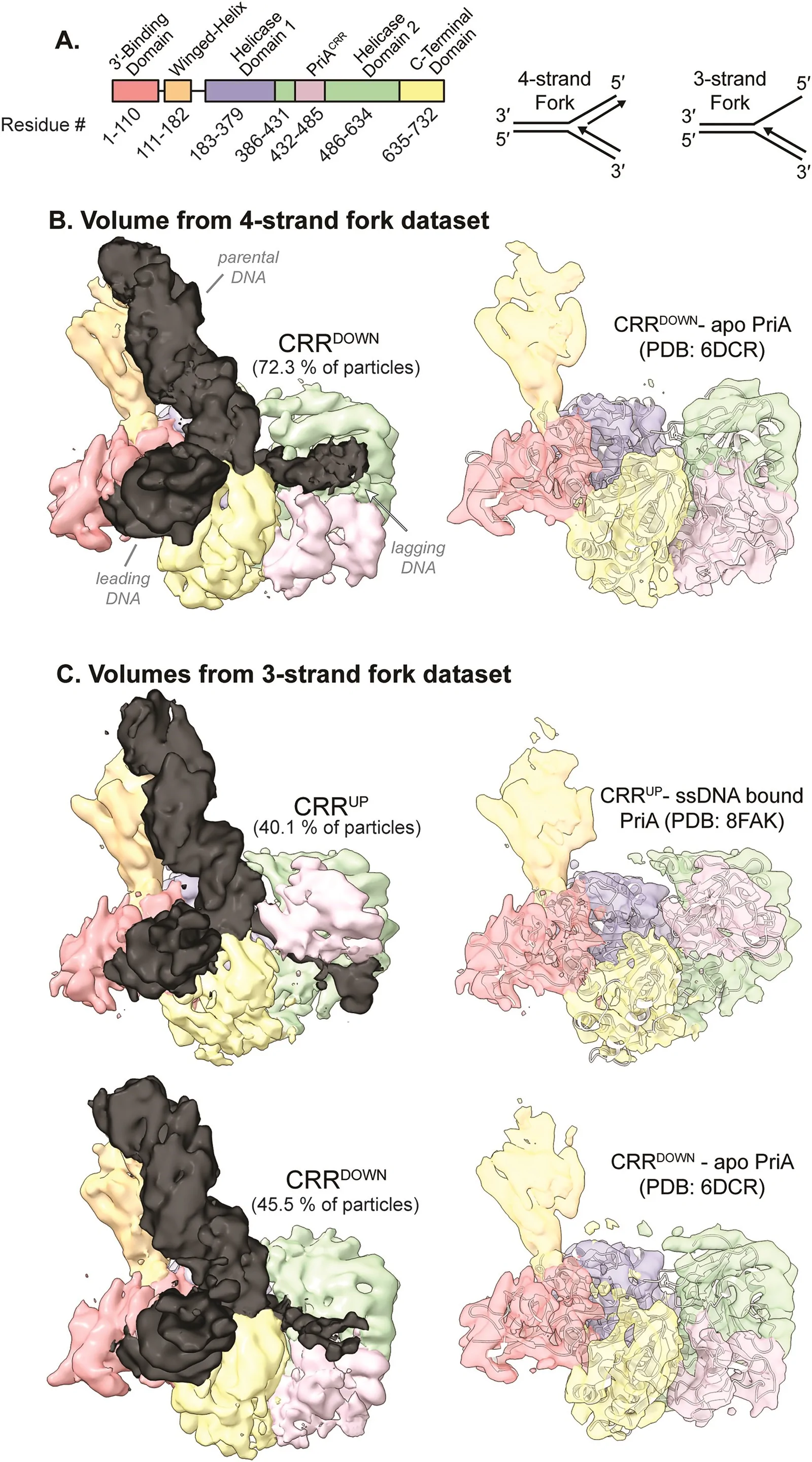
Cryo-EM analysis of *E. coli* PriA^CRR^ dynamics on two structurally unique DNA replication forks. (**A**) Schematics of the functional domains of PriA (3′-Binding domain (salmon), Winged-Helix (tan), Helicase domain 1 (blue), PriA^CRR^ (pink), Helicase domain 2 (green), and C-Terminal domain (yellow)) and the replication forks containing either ssDNA (3-stranded) or dsDNA (4-stranded) lagging strands. Residue numbers indicate PriA domain borders. (**B**) Cryo-EM density map of PriA resembling the CRR^DOWN^ (left) conformation on a 4-strand fork. Density regions are colored by the corresponding PriA domain they represent. Apo PriA (PDB: 6DCR) was fit into the EM density map, demonstrating that the experimental density closely matches the previously observed PriA^CRR^ conformation (right). (**C**) Cryo-EM density maps of PriA resembling either the CRR^UP^ (top left) or CRR^DOWN^ (bottom left) conformations on a 3-strand fork. Density regions are colored by the corresponding PriA domain they represent. PriA from the PriA/PriB/ssDNA lagging strand (PDB: 8FAK) or apo (PDB: 6DCR) structures were fit into the CRR^UP^ (top right) or CRR^DOWN^ (bottom right) EM density maps, respectively, to demonstrate that the experimental density closely matches the previously observed PriA^CRR^ conformations.

Approximately 230,000 particles from the Fork 1 dataset and 280,000 particles from the Fork 2 dataset were classified into good class averages with high-resolution features. *Ab initio* reconstructions for both samples were created from the selected particles and further auto-refined imposing C1 symmetry. Both samples exhibited high levels of conformational heterogeneity and required further processing. The Fork 1 data were subjected to 3D focused classification into 5 classes, focusing on the PriA^CRR^, and were denoised using the Blush denoising program [43] implemented into cisTEM. The Fork 2 data were also subjected to 3D focused classification with Blush denoising but with 10 classes (Supplementary Fig. S2). The resulting maps were classified into either CRR^UP^, CRR^DOWN^, or mixed/unclear, and representative EM density maps are shown in Fig. 1.

Despite the global resolution being measured at higher values (3.5–3.9 Å), the maps exhibited strong preferential orientation as demonstrated by the 3D FSC results (Supplementary Fig. S2) [44]. This led to maps that were of insufficient quality to build molecular models. However, the maps were of sufficient quality to visualize the position of the PriA^CRR^ relative to the rest of PriA and relative to previous structures.

### Zn^2+^ stoichiometry measurements

ZnCl_2_ standards (0–25 µM) were diluted in PAR Buffer (50 mM HEPES–HCl, pH 7.5, 150 mM NaCl, 0.2% SDS, and 0.016 mg/mL Proteinase K) and incubated at 50°C for 20 min at 300 RPM. Samples were supplemented with 50 µM 4-(2-pyridylazo)resorcinol (PAR) (Sigma–Aldrich) and incubated for 1 h at room temperature protected from light. Absorbance values were measured at 500 nm. Data were fit with a linear regression to generate a standard curve in GraphPad PRISM v10.5.0.

WT PriA and PriA CRR variants were buffer exchanged into 50 mM HEPES–HCl, pH 7.5, 150 mM NaCl through an equilibrated desalting column (Bio-Rad). WT PriA and PriA CRR variants (2 µM) were incubated at 50°C for 20 min at 300 RPM in PAR Buffer then incubated with 50 µM PAR for 1 h at room temperature protected from light. Absorbance values were measured at 500 nm. The assay was performed in triplicate for each sample.

### Electrophoretic mobility shift assays 

WT PriA and PriA variants (2 nM) were incubated with ^32^P-labeled Fork 3, 4, 5, 6, 7, or 8 (1 nM) and 0, 10, 50, 100, or 200 nM PriB (monomers) for 15 min at 4°C in 50 mM Tris–HCl, pH 8, 0.1 mg/mL bovine serum albumin (BSA), 2 mM DTT, 5 mM EDTA, and 6% glycerol. Samples were resolved using 4% TBE–PAGE gel for 3 h at 180 V. Gels were exposed to a phosphor screen, imaged using Amersham Typhoon v.2.0.0.2 (Cytiva), quantified using ImageQuant-TL v.11.0 software, and analyzed with GraphPad Prism v10.5.0 software. All electrophoretic mobility shift assays (EMSAs) were performed three or more times, with representative gels shown in figures.

### Helicase assays

WT PriA or PriA variants (0.1, 1, 2, or 5 nM) were incubated PriA or variant with ^32^P-labeled Fork 3, 5, 6, 7, or 8 (1 nM) for 10 min at 37°C in 50 mM HEPES–HCl, pH 8, 40 µg/mL BSA, 2 mM DTT, 4 mM magnesium acetate, and 2 mM ATP. Reactions were terminated with the addition of 20 mM EDTA, 0.5% SDS, 0.2 g/L proteinase K, and 2.5 ng/µL unlabeled 3L-98 oligo and incubated for 20 min at 37°C. DNA loading dye was added, and samples were resolved using 7.5% TBE–PAGE for 3 h at 180 V. Gels were exposed to a phosphor screen, imaged using Amersham Typhoon v.2.0.0.2 (Cytiva), quantified using ImageQuant- TL v.11.0 software, and analyzed with GraphPad Prism v10.5.0 software. All helicase experiments were performed three or more times, with representative gels shown in figures.

PriB-stimulated PriA helicase activity assays were executed as described above, except with 1 nM WT or PriA variants and 0, 0.1, 1, or 10 nM PriB (monomers).

Helicase assays with different lagging-strand gaps were executed as described above, except with 1 nM replication fork and 0.5 nM WT PriA. Assays for WT PriA on gapped forks with 30 nucleotides (nt) of nascent duplex lagging DNA (Forks 16 and 17) were performed with 0.1, 1, 2, or 5 nM WT PriA with 1 nM Fork 16 or Fork 17.

DNA unwinding assays for WT PriA and variants on a DNA replication fork with ss lagging DNA were performed identically to those described above, with 0.1, 1, 2, or 5 nM PriA with 1 nM Fork 4.

### ATP hydrolysis assay

WT PriA or PriA CRR^UP^-destabilized variants (50 nM) were incubated with 0.1–10 000 nM dT_60_ ssDNA in 20 mM HEPES–HCl, pH 8, 50 mM NaCl, 0.1 mg/mL BSA, 1 mM BME, 2 mM phosphoenolpyruvate (PEP), 0.2 mM nicotinamide adenine dinucleotide (NADH), 3 U/mL Pyruvate Kinase (PK), and 4.5 U/mL lactate dehydrogenase (LDH). ATP (1 mM) was added to initiate the reaction. *A*_340 nm_ was monitored for 1 h at 25°C. Data were analyzed with a custom pipeline using Python v.3.10, Pandas v1.4.2, BioPython v1.79, and SciPy v1.8.0. In brief, the absorbance values were normalized by subtracting the baseline absorbance at time zero. The absorbance values were then divided by the molar extinction coefficient of NAHD (ε = 0.00622 μM^−1^ cm^−1^) to convert absorbance into NADH concentration. The rate of NADH consumption was estimated by linear regression over the linear region of the curve and normalized to the concentration of PriA. The resulting data were then further analyzed using GraphPad Prism v10.5.0. Source code for the analysis pipeline is publicly available on GitHub (https://github.com/AYSung/smoltools). Data points are the mean of at least three independent measurements, with error bars representing standard deviation. Data were fit to a Michaelis–Menten kinetic model in GraphPad Prism v10.5.0, with *K*_DNA_ representing the DNA concentration required to reach 50% of the maximum reaction velocity (*V*_max_).

Equation 1: Michaelis–Menten equation for PriA CRR^UP^ with dT_60_ and for all variants on DNA replication fork.


\begin{equation*}{\mathrm{Reaction\ velocity\ }}\left( {{\mathrm{\mu M\ ATP\ mi}}{{\mathrm{n}}}^{ - 1}{\mathrm{\ \mu }}{{\mathrm{M}}}^{ - 1}{\mathrm{\ PriA}}} \right) = \frac{{{{\mathrm{V}}}_{{\mathrm{max}}}{\mathrm{*}}\left[ {{\mathrm{DNA\ }}\left( {{\mathrm{nM}}} \right)} \right]}}{{{{\mathrm{K}}}_{{\mathrm{DNA}}} + \left[ {{\mathrm{DNA\ }}\left( {{\mathrm{nM}}} \right)} \right]}} \\\end{equation*}

For PriA CRR^DOWN^-destabilized variants, experiments were executed identically to those described above, except that 4 mM PEP, 0.4 mM NADH, 6 U/mL PK, and 9 U/mL LDH were used. Data were fit to a one-site binding model using GraphPad Prism v10.5.0, with *V*_max_ representing the maximum reaction velocity, *K*_DNA_ representing the DNA concentration required to reach 50% of *V*_max_, and *V*_min_ representing the minimum reaction velocity measured in the absence of DNA.

Equation 2: One-site binding model for PriA CRR^DOWN^ variants on dT_60_.


\begin{equation*}{\mathrm{Reaction\ velocity\ }}\left( {{\mathrm{\mu M\ ATP\ mi}}{{\mathrm{n}}}^{ - 1}{\mathrm{\ \mu }}{{\mathrm{M}}}^{ - 1}{\mathrm{\ PriA}}} \right) = {{\mathrm{V}}}_{{\mathrm{min}}} + \frac{{\left( {{{\mathrm{V}}}_{{\mathrm{max}}} - {\mathrm{\ }}{{\mathrm{V}}}_{{\mathrm{min}}}} \right){\mathrm{*}}\left[ {{\mathrm{DNA\ }}\left( {{\mathrm{nM}}} \right)} \right]}}{{{{\mathrm{K}}}_{{\mathrm{DNA}}} + \left[ {{\mathrm{DNA\ }}\left( {{\mathrm{nM}}} \right)} \right]}} \\\end{equation*}

For ATP hydrolysis assays performed with a DNA replication fork, WT PriA or PriA variants (5 nM) were incubated with 0.01–100 nM Fork 1 in 20 mM HEPES–HCl, pH 8, 50 mM NaCl, 0.1 mg/mL BSA, 1 mM BME, 2 mM PEP, 0.2 mM NADH, 3 U/mL PK, and 4.5 U/mL LDH. ATP (1 mM) was added to initiate the reaction. *A*_340 nm_ was monitored for 1 h at 25°C. Data were analyzed as described for experiments with dT_60_ using Michaelis–Menten kinetics.

### Mass photometry

Mass photometry experiments were carried out on a TwoMP instrument (Refeyn Inc.), which was allowed to warm up for an hour before any experiments. 20 × 50 mm High Precision cover glass (No. 1.5H of 170 ± 5 µm) coverslips (Azer Scientific) were cleaned sequentially using isopropanol, followed by deionized water three times, then with a final rinse in isopropanol and air-dried on a KimWipe (VWR, Inc.). Any remaining dust was removed using compressed air before usage. Six-well silicone gaskets (cut 2 × 3) (Grace Bio-Labs) were placed onto the top-facing surface of the clean coverslips, then placed onto the oil-immersion objective. MP Binding Buffer (50 mM Tris–HCl, pH 8, 75 mM NaCl, 5 mM EDTA, and 2 mM DTT) was directly loaded into the well to blank and focus the instrument. 10× concentration of protein variants were placed on ice in MP Buffer and diluted to 40 nM PriA variant, 20 nM DNA replication fork, and 100 nM PriB–MBP (monomers) in a final volume of 10 µL in the sample well. Samples were thoroughly mixed prior to starting a 60-s video recording. Data were analyzed against a mass calibration consisting of BSA, Thyroglobulin 1 and Thyroglobulin 2 using Refeyn DiscoverMP software, and nonlinear least squares were fitted with a Gaussian model to quantify the population of molecules within the sample. All experiments were performed in triplicate, with representative data shown in the figure.

### Strains and media

All bacterial strains used in this work are derivatives of *E. coli* K-12. The genotypes and constructions of strains are listed in Supplementary Tables S2–S4. Strains were generated using either linear transformation [45] or P1 transduction [46]. Transformants and transductants were selected on 2% agar plates containing either Luria broth (1% tryptone, 0.5% yeast extract, and 1% sodium chloride) or 56/2 minimal medium supplemented with 0.2% glucose, 0.001% thiamine, 0.02% arginine, 0.005% histidine, 0.02% proline, 0.01% leucine, and 0.01% threonine [46]. When appropriate, ampicillin was used at 50 µg/mL, chloramphenicol at 25 µg/mL, kanamycin at 50 µg/mL, and tetracycline at 10 µg/mL. The cells were purified on the same type of medium on which they were selected and grown at 30 or 37°C. L-Arabinose was used for induction of Lambda Red expression from pKD46 at a final concentration of 0.5% (wt/vol).

### Construction of mutants and transfer to the chromosome

Details for the construction of chromosomal mutations are contained in Supplementary Tables S3 and S4. The constructions followed the same basic strategy. Linear DNA containing the desired *priA* mutation, the appropriate surrounding WT *priA* sequences, and the *cat* gene (just after the *priA* stop codon) was generated through PCR using Phusion polymerase (New England Biolabs) template plasmid DNA synthesized by Twist Biosciences and/or oligonucleotide primers synthesized by Eurofins. These linear DNA fragments were then used to transform strains of *E. coli* carrying a defined partial deletion of the *priA* gene (this varied depending on the location of the codon to be mutated), *dnaC809, 820* (this mutation fully suppresses the defects of a *priA* null mutant), and pKD46. The cells were grown under appropriate conditions to induce the Lambda Red recombination system. Cat^R^ transformants were selected and transformants that replaced the *priA* deletion with the desired *priA* sequences were confirmed by DNA sequencing. P1 transduction was then used to transfer the mutant *priA* gene to a fresh strain (*dnaC^+^*) for characterization.

### Preparation and analysis of cells for microscopy

Cells were grown overnight at 37°C with shaking in 56/2 minimal medium. Three microliters of the culture was then loaded onto a 2% agarose pad prepared from 56/2 minimal medium and low-melting-point agarose. After placing a coverslip over the inoculated agarose pad, slides were incubated for 3 to 4 h at 37°C before imaging. Cells were visualized using a Nikon E600 microscope equipped with automated filter wheels, shutters, a cool LED light source, and an ORCA-ER camera. Phase-contrast and fluorescence images were acquired from 6 to 9 fields of view at a total magnification of 1000. Between 300 and 1500 cells of each strain were analyzed for cell size and fluorescent intensity using the following software: I-Vision (BioVision Technologies), SuperSegger [45], Omnipose [46], MATLAB R2022a (MathWorks, Inc.), and custom MATLAB programs. Statistical analysis of average relative fluorescence intensity (RFI) and cell size was performed with Student’s *t*-test. Cell area was determined from the phase-contrast image. RFI, a measure of SOS expression, was determined from the *sulAp-gfp* promoter fusion, and nucleoid shape was assessed using a *hupA-mcherry* translational fusion. The derivations and characterizations of these molecular tools have been described elsewhere [47, 48]. RFI, reported in Table 1, is the ratio of the average pixel intensity of cells to a defined background area in each image with no cells. The Average Cell Area in Table 1 is the average of the total number of pixels for each cell divided by the total number of cells. The scale of the images is one pixel for 0.0625 microns. The (+) and (−) reported in Table 1 are a qualitative determination of the ability of strains to properly partition their chromosomes. This is determined by considering the cell area and the shapes of the nucleoids. Strains that are (+) are like WT (SS6321), and cells that display poorly partitioning chromosomes are shown in the *priA2::kan* strain (SS7064). Statistical analysis of average RFI and cell area was performed with Student’s *t*-test. For all comparisons in the text, the *P*-value was < 0.001.

**Table 1.** *In vivo* characterization of CRR^UP^-destabilized PriA variants

Strain	*priA*	*priB*	*priC*	Avg. Cell Area (pixels)	Rel. SOS intensity	Chromosome partitioning
SS6321	+	+	+	640	1.1	+
SS7064	*null*	+	+	1670	6.2	−
SS9916	+	*null*	+	710	2.1	+
SS9114	+	+	*null*	630	1.7	+
SS14486	H455A	+	+	710	2.0	+
SS14490	H455A	*null*	+	1210	3.2	−
SS14489	H455A	+	*null*	970	3.0	−/+
SS14467	R427A	+	*+*	630	2.2	+
SS14480	R427A	*null*	*+*	1400	4.6	−
SS14477	R427A	+	*null*	640	2.0	+
SS14469	R428A	+	*+*	580	1.5	+
SS14481	R428A	*null*	+	1600	5.8	−
SS14478	R428A	*+*	*null*	660	1.7	+
SS14468	R427D	*+*	+	1800	7.6	−
SS14470	R428D	*+*	+	2020	8.5	−

*E. coli* strains with mutations to the *priA* allele in either WT, *priB*-null, or *priC*-null backgrounds. Average cell area (number of pixels) was quantified as a measure for elongated cells. Relative SOS Intensity was quantified by the fluorescence of mCherry translationally fused to the *hupA* gene which aids in the visualization of the nucleoid. Nucleoids are indicated as positive “+” for chromosome partitioning and negative “−” for irregular/elongated nucleoids.

## Results

### Replication fork lagging-strand structure influences the position of the PriA^CRR^

PriA comprises six functional domains that collectively form a multi-point binding surface recognizing the parental, leading-, and lagging-strand arms of DNA replication forks (Fig. 1A) [19–22, 31, 49, 50]. Prior structural analysis of PriA/PriB/replication fork and PriA/PriB/DnaT/replication fork complexes revealed that PriA binding to a replication fork with a ssDNA lagging strand repositioned the PriA^CRR^ by rotating the domain ∼80° relative to its position in apo PriA (Supplementary Fig. S3 and Supplementary Video S1) [21, 35]. This conformational change creates a pore encircling the lagging strand. The pore width is sufficient to accommodate ssDNA but not dsDNA, suggesting that this motion would not occur with replication forks containing dsDNA lagging strands. To test if a DNA replication fork alone could trigger a PriA domain rearrangement in the absence of other preprimosome proteins, we used cryoEM to analyze PriA bound to replication forks containing lagging strands that were either ssDNA (15 bases) or dsDNA (with a short 5-base gap between the fork junction and the nascent lagging strand) (Fig. 1 and Supplementary Table S1).

The resulting maps showed that the PriA^CRR^ is conformationally heterogeneous compared to the rest of PriA. Dividing the data into multiple classes using 3D classification and focusing on the PriA^CRR^ enabled the determination of low-resolution structures (∼3.5–6 Å) where PriA^CRR^ is either in a downward (CRR^DOWN^) or upward (CRR^UP^) position relative to the helicase core and the parental duplex of the fork (Fig. 1B and C, and Supplementary Figs S1 and S2). These positions closely match those seen in previous apo PriA structures (CRR^DOWN^) or the PriA/PriB/replication fork and PriA/PriB/DnaT/replication fork structures, respectively [19–21, 35]. Map qualities were sufficient for rigid-body docking of previously characterized PriA structures, but not for *de novo* molecular modeling [19–21].

The PriA^CRR^ position was best defined when bound to the DNA replication fork with a dsDNA lagging strand, where 72.3% of the total particles were classified into a volume that closely resembled the PriA CRR^DOWN^ state observed in previous apo structures (Fig. 1B) [19, 20]. The remaining particles were classified into a volume with no clear density for the PriA^CRR^, highlighting the inherent flexibility of this domain. In contrast, when bound to a ssDNA lagging strand, the PriA^CRR^ could be found in states resembling either the CRR^UP^ or CRR^DOWN^ positions seen in previous structures (Fig. 1C) [19–21]. Here, 40.1% and 45.5% of the particles were classified into volumes resembling the CRR^UP^ and CRR^DOWN^ states, respectively. The remaining particles lacked clear PriA^CRR^ density. These data suggest that, while the PriA^CRR^ is conformationally heterogeneous, a dsDNA lagging strand strongly biases PriA toward the CRR^DOWN^ state, whereas the ssDNA lagging strand allows sampling of both conformations. Since the PriA^CRR^ was uniformly in the CRR^UP^ state in the PriA/PriB/replication fork and PriA/PriB/DnaT/replication fork structures [21, 35], PriB docking appears to lock PriA into the CRR^UP^ conformation.

### Eleven bases of lagging-strand ssDNA are required for PriB association with PriA

**Figure 2.**
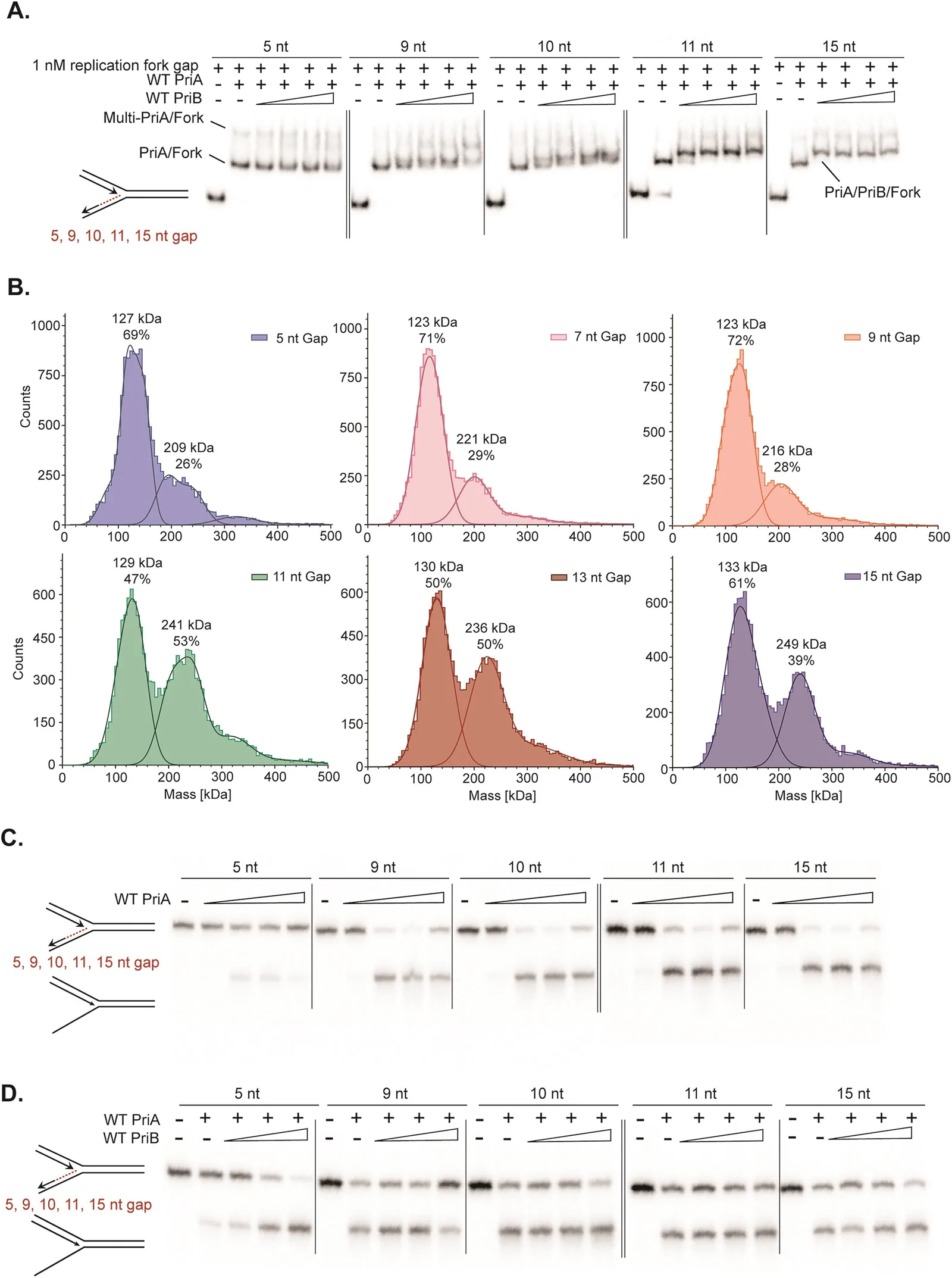
PriA/PriB/replication fork complex formation requires 11 bases of lagging-strand ssDNA. (**A**) PriB interaction with PriA/replication fork complexes containing ssDNA gaps [5-, 9-, 10-, 11-, and 15-nucleotides (nt)] between the fork junction and duplexed lagging strand. Complexes formed between replication forks (1 nM), PriA (2 nM), and PriB (0, 10, 50, 100, or 200 nM, monomers) were resolved using native TBE–PAGE. Single lines separate lanes from a single gel, and double lines indicate where multiple gels are aligned in the figure. (**B**) Mass photometry analysis of PriB–MBP interaction with PriA bound to replication forks containing 5-, 7-, 9-, 11-, 13-, or 15-base ssDNA lagging-strand gaps. PriA (40 nM), PriB–MBP (100 nM, monomers), and replication forks (20 nM) were co-incubated and mass distribution was recorded. Calculated molecular weights are: PriA (81 kDa), replication fork (∼55 kDa), PriB–MBP (dimer, 116 kDa), PriA/replication fork (∼130 kDa), and PriA/PriB/replication fork (∼240 kDa). (**C**) PriA unwinding of replication forks containing 5-, 9-, 10-, 11-, and 15-base ssDNA lagging-strand gaps. Replication forks (1 nM) and PriA (0.1, 1, 2, or 5 nM) were co-incubated with ATP. DNA unwinding products were resolved using native TBE–PAGE. (**D**) PriB stimulation of PriA DNA helicase activity on replication forks with 5-, 9-, 10-, 11-, and 15-base ssDNA lagging-strand gaps. Replication forks (1 nM), PriA (0.5 nM), and PriB (0, 0.1, 1, or 10 nM, monomers) were co-incubated with ATP. DNA unwinding products were resolved using native TBE–PAGE.

PriB interacts with PriA/replication fork DNA complexes by binding to both lagging-strand ssDNA and a site on PriA^CRR^ that is accessible only in the CRR^UP^ state [21, 26]. However, the length of ssDNA needed for stable PriB docking is unknown. To estimate this length, PriB binding to PriA/replication fork complexes with ssDNA lagging-strand gaps ranging from 5- to 15-bases was assessed using an EMSA (Fig. 2A) [19, 21, 29].

Consistent with prior studies [19, 21, 26, 30, 31, 39, 51], PriA bound radiolabeled replication fork DNA with a 5-base gap between the fork junction and the dsDNA lagging strand, reducing its mobility in a gel (Fig. 2A and Supplementary Fig. S4). Titration of PriB did not alter the gel mobility of the complex, indicating that PriB could not stably interact with PriA bound to a replication fork with a 5-base gap. In contrast, PriB readily shifted the mobility of the complex formed between PriA and a replication fork with a 15-base gap (Fig. 2A and Supplementary Fig. S4), indicating that stable PriB binding required a lagging-strand ssDNA gap size of between 5 and 15 bases.

To better understand the minimal ssDNA gap size required for stable PriB interaction with PriA/replication fork complexes, DNA substrates with intermediate gaps were tested. When the lagging-strand gap size was 9 or 10 bases, the addition of PriB led to smeared, super-shifted bands (Fig. 2A). This behavior is consistent with PriB dissociating from the PriA/replication fork during electrophoresis, and with a weaker PriB interaction than that observed with longer gapped DNA. However, when the ssDNA gap size was increased to 11 bases, a clear PriB-dependent super-shifted band appeared, indicating stable PriA/PriB/replication fork complex formation (Fig. 2A and Supplementary Fig. S4). These results align with the PriA/PriB/replication fork cryo-EM structure, which shows PriA occluding nine bases of ssDNA and PriB binding to the PriA^CRR^ in the CRR^UP^ position and to the ssDNA bases that are 10 and 11 nucleotides away from the fork junction [21]. Although DnaT also requires the CRR^UP^ conformation to bind PriA [35], our analysis was restricted to PriB docking due to anticipated complications with DnaT filamentation and contributions of PriB in stabilizing DnaT inclusion in the preprimosome complex.

We used mass photometry as a complementary method to examine PriA/PriB/replication fork complex formation. PriA and PriB–MBP dimer (used to increase mass and improve detection, individual mass photometry profiles shown in Supplementary Fig. S5) was combined with replication forks containing 5-, 7-, 9-, 11-, 13-, or 15-base lagging-strand gaps. For the 5-, 7-, and 9-base gap replication forks, the PriA/DNA complex was the dominant species (69%–72%, ∼125 kDa), with 209–221 kDa peaks accounting for the remainder (Fig. 2B). The minor population could arise from PriB–MBP binding to the PriA/DNA replication fork complex and/or from higher-mass species detected with PriB–MBP (Supplementary Fig. S5). Further investigation using a PriA variant known to be deficient in PriB binding (PriA^CRR^ residues D438A, S481A, and T482A) [21] showed that, when bound to a replication fork with ssDNA lagging strand, the same minor ∼215 kDa peak appeared, indicating the minor peak is not due to specific docking onto PriA^CRR^ (Supplementary Fig. S6).

Increasing the lagging-strand gap to 11 or more bases caused a significant change in the molecular mass distribution (Fig. 2B). PriA and PriB–MBP binding to these substrates formed a prominent ∼240 kDa PriA/PriB–MBP dimer/DNA complex, accounting for 39%–53% of all species (Fig. 2B). These results independently confirm the EMSA findings, demonstrating that an 11-base lagging-strand gap is required for PriB to bind stably to the PriA/replication fork complex. Since PriB docking requires access to its binding site on PriA in the CRR^UP^ position and to two bases of ssDNA, these data are consistent with a lagging-strand gap of nine or more bases being required to promote formation of the PriA CRR^UP^ conformation and two or more bases of ssDNA emerging from the PriA/DNA complex for stable PriB docking [21].

### Effects of PriA^CRR^ mobility and PriB on PriA helicase activity

Prior studies have suggested that a β hairpin within the PriA^CRR^ acts as a wedge to promote lagging-strand duplex DNA unwinding when PriA is in the CRR^DOWN^ conformation [29, 30, 52]. In contrast, PriA has also been shown to unwind duplex DNA when long ssDNA gaps are present [15], which are substrates that PriA can bind in either the CRR^UP^ or CRR^DOWN^ conformation based on our observations. Whether both PriA^CRR^ positions support helicase activity is not known.

To examine how PriA^CRR^ domain dynamics affect helicase activity, we first assessed the ability of PriA to unwind replication forks with varying lagging-strand gap sizes. As previously observed [15, 26, 53], PriA exhibited low helicase activity on a replication fork containing a 5-base lagging-strand gap (Fig. 2C and Supplementary Fig. S7A). However, when the gap size increased to 9 or more bases, the unwinding activity of PriA became more efficient. This suggests that PriA^CRR^ mobility enhances helicase efficiency and that the CRR^UP^ conformation of PriA supports more robust helicase activity than the CRR^DOWN^ conformation.

A potential confounding factor in this experiment is that, because the total length of the lagging strand remained constant across the different substrates, longer-gapped substrates have shorter duplex regions. This could influence the results if the processivity of PriA is challenged by longer duplex structures. To evaluate this possibility, PriA helicase activity was measured on replication fork substrates with either 5- or 15-base gaps that included the same duplex length (30 bp) (Supplementary Fig. S7B and C, and Supplementary Table S1). PriA unwound the 15-base-gap substrate far more efficiently than the 5-base-gap substrate. This result indicates that the lagging-strand gap size, and not the duplex length, dictates helicase efficiency. These findings align with a prior study showing that DNA unwinding by PriA increases with larger lagging-strand gap sizes [15] and suggest that PriA helicase activity is weak when the enzyme is in the CRR^DOWN^ conformation, as seen with a 5-base lagging-strand gap, but is robust when PriA is in the CRR^UP^ conformation.

We next investigated whether PriB differentially affects PriA-mediated DNA unwinding using replication forks with ssDNA gaps of different sizes. As observed previously [26], PriB enhanced PriA DNA unwinding of the 5-base lagging-strand gap replication fork in a concentration-dependent manner (Fig. 2D and Supplementary Fig. S7D). In contrast, adding PriB had little to no effect on PriA DNA unwinding of forks with larger gaps (Fig. 2D and Supplementary Fig. S7D). These findings highlight an apparent paradox: helicase stimulation (Fig. 2D and Supplementary Fig. S7D) was inversely related to stable PriB binding to PriA/DNA (Fig. 2A and B) even though PriB must bind to the PriA^CRR^ to stimulate its helicase activity [21]. This contradiction suggests a complex relationship between PriA^CRR^ and PriB during lagging-strand unwinding, which is investigated further herein.

### PriA variants designed to impair interdomain contacts stabilizing the CRR^UP^ state and interactions with lagging-strand ssDNA disrupt PriB binding and PriA helicase activity

**Figure 3.**
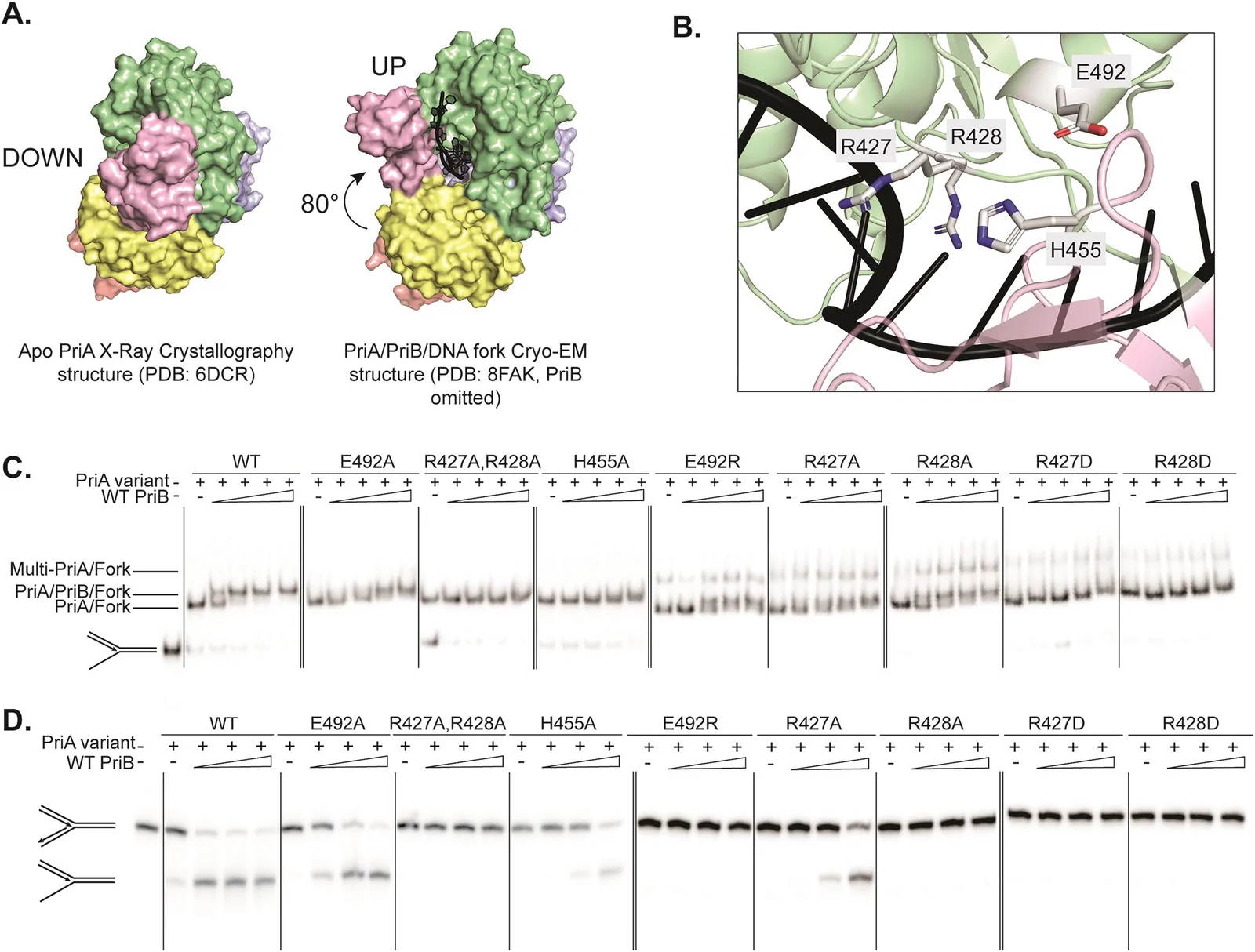
CRR^UP^-destabilized PriA variants impair helicase activity and stable PriB binding. (**A**) Structures depicting *E. coli* PriA in CRR^DOWN^ and CRR^UP^ conformations [19, 21]. Arrow illustrates rotation of the PriA^CRR^ into CRR^UP^ orientation. (**B**) Interface formed between helicase domain 2 (green) and PriA^CRR^ (pink) or ssDNA in the CRR^UP^ conformation. (**C**) PriB binding to PriA/replication fork complex. Complexes formed by replication fork DNA (1 nM), WT PriA or CRR^UP^ variants (2 nM), and PriB (0, 10, 50, 100, or 200 nM, monomers) were resolved using native TBE–PAGE. Single lines separate lanes from a single gel, and double lines indicate where multiple gels are aligned in the figure. (**D**) PriB stimulation of PriA DNA helicase activity. Replication fork (1 nM), WT PriA or CRR^UP^ variants (1 nM), and PriB (0, 0.1, 1, and 10 nM, monomers) were incubated with ATP. DNA unwinding products were resolved using native TBE–PAGE.

The PriA CRR^DOWN^-to-CRR^UP^ conformational change exposes the PriB-binding site and encloses lagging-strand ssDNA [21] (Fig. 3A). We hypothesized that sequence changes in PriA residues that form interdomain contacts in the CRR^UP^ conformation or that interact with lagging-strand ssDNA within the PriA pore could alter its ability to bind PriB and possibly other activities, providing insights into the roles of PriA^CRR^ dynamics. The CRR^UP^ conformation of the PriA^CRR^ is stabilized by the side chain of E492 in helicase domain 2 (PriA^HD2^), which forms hydrogen bonds with the backbone amides of Q456 and A457, and by the side chain of H455 within the PriA^CRR^ interacting with D513 in PriA^HD2^ (Supplementary Fig. S8A). Additionally, residues R427 and R428, which are part of helicase motif IV in PriA^HD2^ [19, 20], interact with the lagging-strand template ssDNA within the PriA pore (Supplementary Fig. S8B). Eight PriA variants predicted to destabilize the CRR^UP^ conformation by disrupting the interface between PriA^HD2^ and PriA^CRR^ (H455A, E492A, and E492R) or with lagging-strand ssDNA (R427A, R427D, R428A, R428D, and R427A/R428A) were purified. These variants, referred to as CRR^UP^-destabilized variants (Fig. 3B), were purified and tested for biochemical activity. Each of the CRR^UP^-destabilized PriA variants bound two zinc ions as expected, indicating that the sequence changes did not disrupt folding or zinc binding within the PriA^CRR^ of the variant proteins (Supplementary Fig. S9).

The ability of the CRR^UP^-destabilized PriA variants to form complexes with PriB was first measured to determine whether the mutations compromised PriB binding, which could indicate a defect in forming the CRR^UP^ state required to expose the PriB-binding site. WT PriA and each of the CRR^UP^-destabilized variants shifted the gel mobility of a replication fork with a ssDNA lagging strand, indicating that each variant was properly folded and still could bind DNA (Fig. 3C). The lack of DNA-binding defects in the R427 and R428 variants is likely because 3′-nascent leading-strand end binding is the dominant interaction surface used by PriA [54], which would mask potential disruptions in variant binding to the lagging strand.

When increasing concentrations of PriB were added to the PriA/DNA complexes, WT PriA/DNA formed a stable complex with PriB, illustrated as a super-shifted species in the gel, but the R427A/R428A, R427D, R428D, and H455A PriA variants did not (Fig. 3C and Supplementary Fig. S10A). PriA CRR^UP^-destabilized variants R427A, R428A, E492A, and E492R showed smeared bands at high PriB levels, suggesting more modest PriB binding defects (Fig. 3C and Supplementary Fig. S10A). These results support roles for PriA residues R427, R428, H455, and E492 in stabilizing the CRR^UP^ conformation required for PriB binding.

DNA unwinding assays were used to determine whether destabilizing the CRR^UP^ conformation affects PriB-stimulated PriA helicase activity. WT PriA unwound the lagging strand of a replication fork with a 5-base lagging-strand gap inefficiently, and the addition of PriB significantly stimulated activity (Fig. 3D and Supplementary Fig. S10B–D). Unlike WT PriA, each of the CRR^UP^-destabilized variants lacked DNA unwinding activity in the absence of PriB (Fig. 3D, and Supplementary Fig. S10B and C). This was anticipated for the R427 and R428 variants, which have sequence changes at known helicase motif residues, but not for the H455 and E492 variants, which form interdomain interactions and are not within helicase motifs.

PriB restored helicase activity in the E492A PriA CRR^UP^-destabilized variant, and, to a more modest extent, in the H455A and R427A variants (Fig. 3D and Supplementary Fig. S10D). PriB did not stimulate helicase activity in the E492R variant, despite weak binding observed in gel shift analysis. Variants with mutations in motif IV residues R427A/R428A, R428A, R427D, and R428D, which failed to form complexes with PriB (Fig. 3C), were not activated by PriB (Fig. 3D and Supplementary Fig. S10D). Overall, nearly all CRR^UP^ conformational variants had either reduced or eliminated DNA unwinding activity.

### PriA variants designed to impair interdomain contacts stabilizing the CRR^DOWN^ state support normal PriB binding and PriA helicase activities

**Figure 4.**
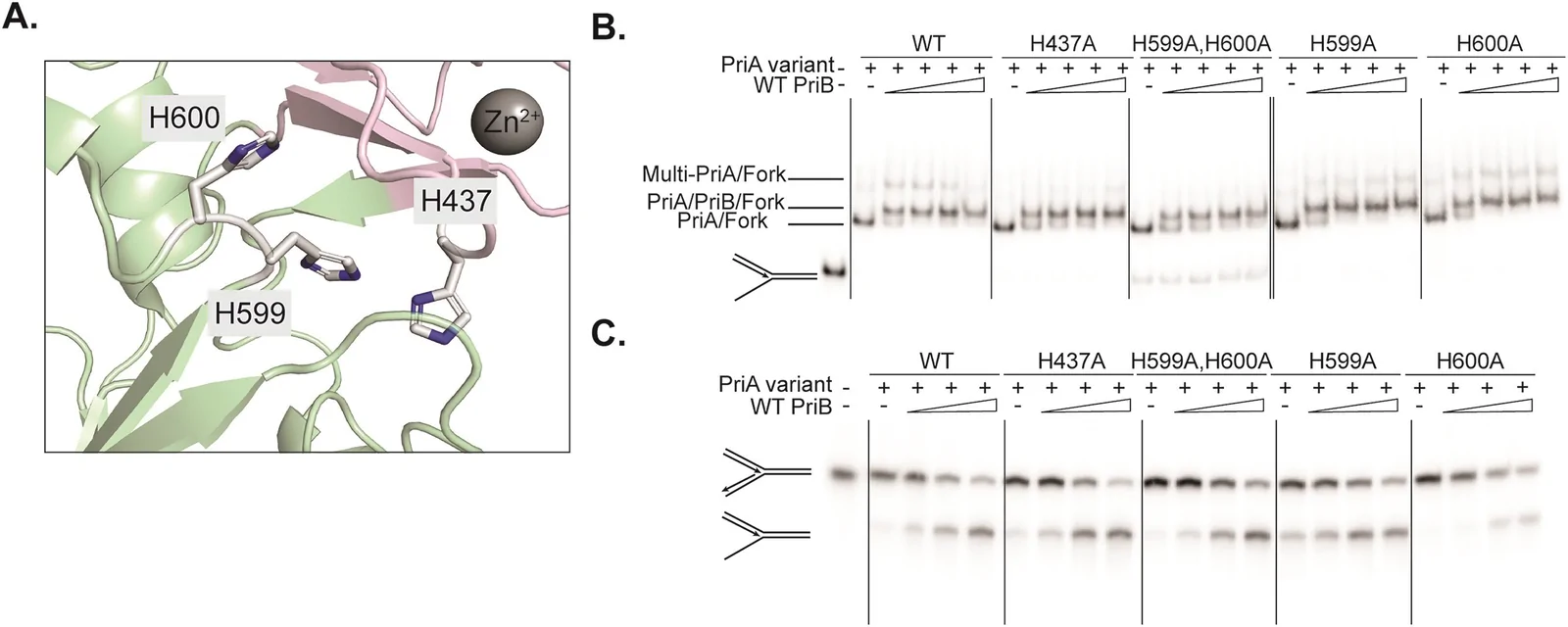
CRR^DOWN^-destabilized PriA variants do not impair PriB binding. (**A**) Interface formed between PriA helicase domain 2 (green) and PriA^CRR^ (pink) in the CRR^DOWN^ conformation [19]. (**B**) PriB binding to PriA/replication fork complex. Complexes formed by replication fork (1 nM), WT PriA or CRR^DOWN^ variants (2 nM), and PriB (0, 10, 50, 100, or 200 nM, monomers) were resolved using native TBE–PAGE. Single lines separate lanes from a single gel, and double lines indicate where multiple gels are aligned in the figure. (**C**) PriB stimulation of PriA DNA helicase activity. Replication fork (1 nM), WT PriA or CRR^DOWN^ variants (1 nM), and PriB (0, 0.1, 1, 10 nM, monomers) were incubated with ATP. DNA unwinding products were resolved using native TBE–PAGE. Single lines separate lanes from a single gel, and double lines indicate where multiple gels are aligned in the figure.

The CRR^DOWN^ conformation is stabilized by several electrostatic interactions: H600 in PriA^HD2^ binding G440 in PriA^CRR^, H437 in PriA^CRR^ binding Q397 in PriA^HD2^, and H599 in PriA^HD2^ interacting with H437 in PriA^CRR^ via their side chains, with additional contacts between the backbone carbonyl of H599 and the side chain of K395 in PriA^HD2^ (Fig. 4A and Supplementary Fig. S8C). Four CRR^DOWN^-destabilized variants (H437A, H599A, H600A, and H599A/H600A) were purified and tested for biochemical activity. Each of the CRR^DOWN^-destabilized PriA variants bound two zinc ions per protein, indicating proper folding (Supplementary Fig. S9).

Binding of the PriA CRR^DOWN^-destabilized variants to replication fork DNA and PriB closely matched that of WT PriA (Fig. 4B and Supplementary Fig. S11A), indicating that the sequence changes did not impact DNA binding or PriB interaction. In DNA unwinding assays performed in the absence of PriB, the H437A and H599A variants showed comparable helicase activity to that of WT PriA, whereas H600A and H599A/H600A showed weak DNA unwinding (Supplementary Fig. S11B and C). The helicase activity of each PriA CRR^DOWN^-destabilized variant could be stimulated by PriB, with stimulation of the H600A variants being somewhat more modest (Fig. 4C and Supplementary Fig. S11D). Compared with the results for the CRR^UP^-destabilized variants, destabilization of the CRR^DOWN^ interface had minimal effects on PriB binding and PriB-stimulated helicase activity.

### PriA CRR^UP^- and CRR^DOWN^-destabilized variants differentially affect ATP hydrolysis

**Figure 5.**
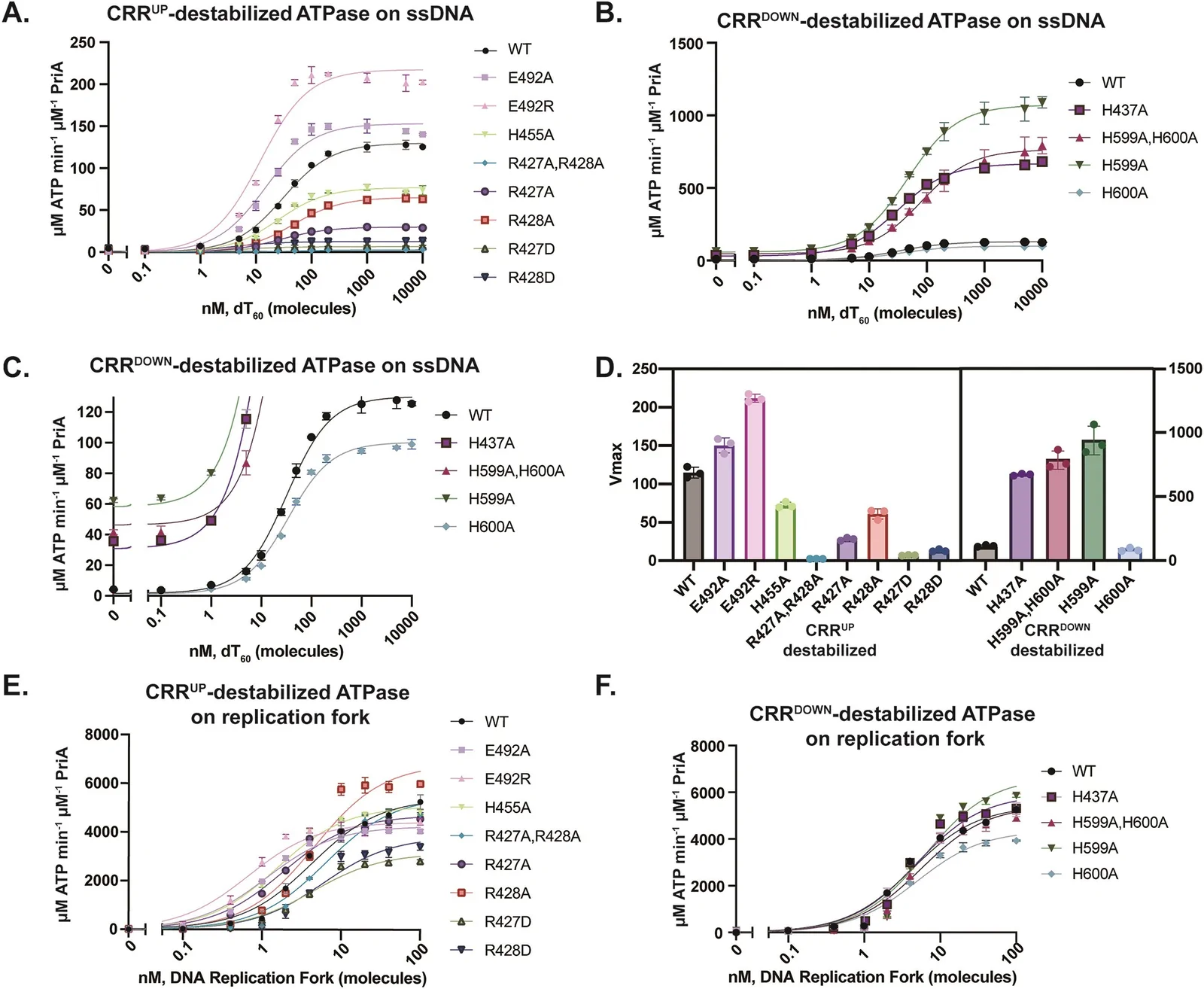
Effects of CRR^UP^- and CRR^DOWN^-destabilized variants on DNA-dependent ATP hydrolysis. (**A**) dT_60_-dependent ATP hydrolysis activity of PriA CRR^UP^-destabilized variants. (**B**) dT_60_-dependent ATP hydrolysis of PriA CRR^DOWN^-destabilized variants. (**C**) Close-up view of dT_60_-dependent ATP hydrolysis illustrating DNA-independent activity of PriA CRR^DOWN^-destabilized variants. (**D**) Comparison of *V*_max_ values for PriA CRR^UP^- (left) and CRR^DOWN^-destabilized (right) variants with dT_60_. Data depict the mean of three replicates with standard deviation error bars. (**E**) Replication fork-dependent ATP hydrolysis activity of WT PriA and CRR^UP^-destabilized variants. (**F**) Replication fork-dependent ATP hydrolysis activity of WT PriA and CRR^DOWN^-destabilized variants.

To examine whether variants designed to destabilize the CRR^UP^ and CRR^DOWN^ PriA conformations influence its ATPase functions, DNA-dependent ATP hydrolysis activities were measured. As previously observed [22], WT PriA is a weak ATPase without DNA (4.3 ± 0.21 min^−1^) but is stimulated 30-fold by the addition of dT_60_ ssDNA (Fig. 5). dT_60_-stimulated activity reaches 50% saturation (*K*_DNA_) with 31 ± 4.4 nM ssDNA.

The PriA CRR^UP^-destabilized variants exhibited a range of ssDNA-dependent ATPase profiles (Fig. 5A and D). Variants R427A/R428A, R427D, and R428D exhibited very low ATPase activity that was not stimulated by dT_60_ (Fig. 5A and D), consistent with roles for these helicase motif residues in linking ssDNA binding to ATPase activity. Variants R427A, R428A, and H455A had 2- to 4-fold lower maximum hydrolysis rates than WT and had *K*_DNA_ values very similar to those of WT PriA, consistent with DNA-binding affinity remaining unaffected by the sequence changes, but with a modest reduction in ATPase function. E492A and E492R displayed slightly higher hydrolysis rates than WT, with similar *K*_DNA_ values (Fig. 5A and D).

Three of the four CRR^DOWN^-destabilized PriA variants (H599A/H600A, H599A, and H437A) showed hyperactive ATP hydrolysis profiles, while the fourth (H600A) nearly matched WT PriA (Fig. 5B and D). The maximum dT_60_-stimulated ATPase rates for H599A/H600A, H599A, and H437A ranged from 660 to 1, 100 min^−1^, representing a 6- to 10-fold increase over WT PriA. The *K*_DNA_ values for the CRR^DOWN^-destabilized PriA variants were similar to WT PriA, consistent with DNA-binding affinity being unaffected by the sequence changes. Interestingly, H599A/H600A, H599A, and H437A variants also showed 10- to 17-fold higher ATPase activity in the absence of DNA (Fig. 5C), suggesting that destabilizing the CRR^DOWN^ conformation, and possibly inducing PriA^CRR^ dynamics and formation of the CRR^UP^ conformation, could stimulate ATPase activity.

We next examined whether destabilizing the CRR^UP^ or CRR^DOWN^ PriA conformations affected ATP hydrolysis stimulated by a DNA replication fork that contained duplex parental and leading strand DNA, along with a lagging-strand ssDNA arm. WT and variant PriAs do not unwind this substrate (Supplementary Fig. S12 and Supplementary Table S1), which allows measurement of ATPase activity without complications from DNA unwinding. WT PriA is stimulated >800-fold when the DNA replication fork is added (Fig. 5E and F), which is 41-fold higher than its stimulation with dT_60_. The *K*_DNA_ of the replication fork substrate was 3.5 ± 1.2 nM, approximately 9-fold lower than that with ssDNA, consistent with higher-affinity binding to the replication fork structure, as has been observed previously [55–58].

Interestingly, replication fork DNA stimulated ATPase activity of all the CRR^UP^-destabilized variants (Fig. 5E). This contrasts sharply with the absence of ATP hydrolysis observed in CRR^UP^-destabilized variant R427A/R428A, and the significantly reduced hydrolysis in variants R427A, R428A, R427D, and R428D with ssDNA (Fig. 5A). ATP hydrolysis in the CRR^DOWN^-destabilized variants showed similar rates and *K*_DNA_ values as WT (Fig. 5F). Differences in the ATPase profiles with dT_60_ and replication fork DNA may reflect engagement of multiple domains with the replication fork structure, which could enhance positioning and the local concentration of lagging-strand ssDNA along the ssDNA-binding surface of the helicase/ATPase domain of PriA.

### PriA CRR^UP^-destabilized variants impair replication restart *in vivo*

*priA*-null *E. coli* cells show poor viability, elevated expression of SOS DNA-damage genes, deficiencies in DNA recombination and repair, impaired chromosome partitioning, and SOS-independent cell filamentation [13, 59–61]. PriA helicase and ATPase activities are not required for a functional PriA/PriB pathway, while mutations in the PriA^CRR^ can disrupt this pathway [16, 21, 52, 61]. In contrast, PriA helicase and ATPase functions are required for the backup PriA/PriC pathway [61].

To explore whether the PriA^CRR^ position variants affect replication restart *in vivo*, we introduced selected CRR^UP^-destabilized mutations (R427A, R428A, R427D, R428D, H455A) and CRR^DOWN^-destabilized mutations (H437A, H600A) into the *priA* gene in *E. coli*. The effects of these mutations were studied in three genetic contexts: (i) a wild-type background, (ii) *priB*-null cells (which route replication restart through PriA/PriC or PriC pathways), or (iii) *priC*-null cells (which rely exclusively on the PriA/PriB pathway). Genomic instability was evaluated by measuring SOS induction, cell filamentation, and chromosome partitioning deficiency.

In an otherwise wild-type genetic background, the CRR^UP^/motif IV R427D and R428D mutations led to increased genomic instability resembling that of the *priA*-null control strain (Table 1 and Supplementary Fig. S13). These mutant cells exhibited 7- to 8-fold induction of SOS, elongated cell morphology, and a chromosome partition defect. Alanine substitution of the same residues (R427A and R428A) resulted in more subtle phenotypic differences, with 2-fold SOS induction. These phenotypes are far stronger than those of an ATPase-dead PriA [61], suggesting that the motif IV charge-reversal variants are toxic.

In *priB*-null cells, PriA motif IV mutants R427A and R428A displayed genomic instability phenotypes similar to a *priA*-null (Table 1 and Supplementary Fig. S13), whereas R427D and R428D mutations could not be made (suggesting the mutations were lethal in *priB*-null cells). The H455A CRR^UP^-destabilized variant also displayed significantly elevated SOS, elongated cell morphology, and chromosome partition defects. In contrast, the PriA H437A CRR^DOWN^-destabilized mutant phenotypes matched those of *priA^+^* cells, whereas PriA H600A mutant cells displayed modest genomic instability phenotypes (Supplementary Table S5). This behavior aligns well with prior genetic studies: *priB*-null cells rely heavily on the PriA/PriC restart pathway, which requires a functional PriA ATPase/DNA helicase [61], and each of the CRR^UP^-destabilized variants lacked helicase activity *in vitro* in the absence of PriB whereas the H437A CRR^DOWN^-destabilized variant had helicase activity, and the H600A variant had lower helicase levels (Figs 3C and 4C, and Supplementary Figs S10 and S11). These results suggest that impairing ssDNA binding within the PriA pore or destabilizing the CRR^UP^ conformation significantly affects PriA function during replication restart.

In *priC*-null cells, only one mutant, encoding the H455A CRR^UP^-destabilized variant, showed an increase in cell size, SOS induction, and a modest chromosome partitioning defect (Table 1 and Supplementary Fig. S13). Prior to this result, three other *priA* mutants had been found that show additive effects with *priC* deletion: *priA301* (C479Y), *del(priA)340*, and *priA355* (D438A, T482A, and H483A), all of which alter the PriA^CRR^, and lead to similarly modest effects when coupled with a *priC* deletion [14, 21, 61]. These results suggest that destabilization of the CRR^UP^ conformation or blockage of PriB docking had a more detrimental impact on the PriA/PriB pathway than loss of helicase activity.

## Discussion

While the cellular mechanisms that control DNA replication initiation in bacteria are well established, how DNA replication restart is regulated is less clear. Here, we have examined the structural mechanisms governing replication fork recognition by *E. coli* PriA, the protein that initiates replication restart, and the effects of PriA sequence changes designed to alter PriA^CRR^ domain dynamics observed when bound to DNA replication forks. The results link the structural flexibility of PriA to its biochemical and cellular activities, pointing to replication fork recognition and remodeling as key steps that regulate DNA replication restart.

Cryo-EM analysis revealed that the position of the PriA^CRR^ domain is influenced by the structure of the lagging strand in a DNA replication fork. When bound to a replication fork with a ssDNA lagging strand, two main positions of the PriA^CRR^ domain were observed. One aligned with that of apo PriA (CRR^DOWN^) [19], while the other matched PriA in the PriA/PriB/DNA and PriA/PriB/DnaT/DNA complexes (CRR^UP^) [21, 35]. The two positions differ by an ∼80° rotation of the PriA^CRR^, enabling PriA to encircle the ssDNA lagging strand in the latter state while also exposing the binding sites for downstream primosome protein docking. Both forms were nearly equally represented among the particles analyzed, with a smaller subset showing no apparent density for PriA^CRR^. These findings suggest that the PriA^CRR^ is a hypermobile structural element when bound to a replication fork with a sufficiently long ssDNA lagging strand. Interestingly, the PriA^CRR^ was exclusively in the CRR^UP^ conformation and was resolved at high resolution in the PriA/PriB/DNA and PriA/PriB/DnaT/DNA structures [21, 35]. This is consistent with PriB selectively docking onto the PriA/replication fork complex when the CRR^UP^ conformation is present [21], restricting the complex to a single conformation.

In contrast, when PriA was bound to a replication fork with a dsDNA lagging strand, the position of the PriA^CRR^ domain resembled that of apo PriA in most particles [19, 20] and was not resolved in the remaining classes of particles. In this scenario, the domain might be transiently sampling conformations similar to the CRR^UP^ state, but it is unable to close around the lagging strand because the diameter of the binding pore is too small to accommodate dsDNA. Such size selectivity by the PriA pore has been proposed [21]. Unwinding of a duplex lagging strand by PriA could generate ssDNA required for the protein to adopt the CRR^UP^ conformation.

After recognition of the replication fork and remodeling of the lagging strand by PriA, the next step in restarting replication is the recruitment of PriB [39]. Stable binding of PriB requires both a ssDNA lagging strand and a docking surface on the PriA^CRR^ that is exposed only in the CRR^UP^ conformation [21, 26, 30]. In this conformation, PriA traps nine bases of lagging-strand ssDNA in a pore, with PriB binding at least two bases that extend from the PriA pore [21]. A previous study, confirmed here, showed that PriB binding is blocked when the lagging strand has a five-base ssDNA gap [26] (Fig. 2). Finer mapping in our current study indicates that stable PriB docking requires PriA to bind a replication fork with at least an 11-base ssDNA gap. Collectively, these data support a model in which formation of the CRR^UP^ conformation requires nine ssDNA bases, and PriB docking needs at least two nucleotides of ssDNA from the lagging strand to exit the PriA pore, as observed in the PriA/PriB/DNA structure [21].

**Figure 6.**
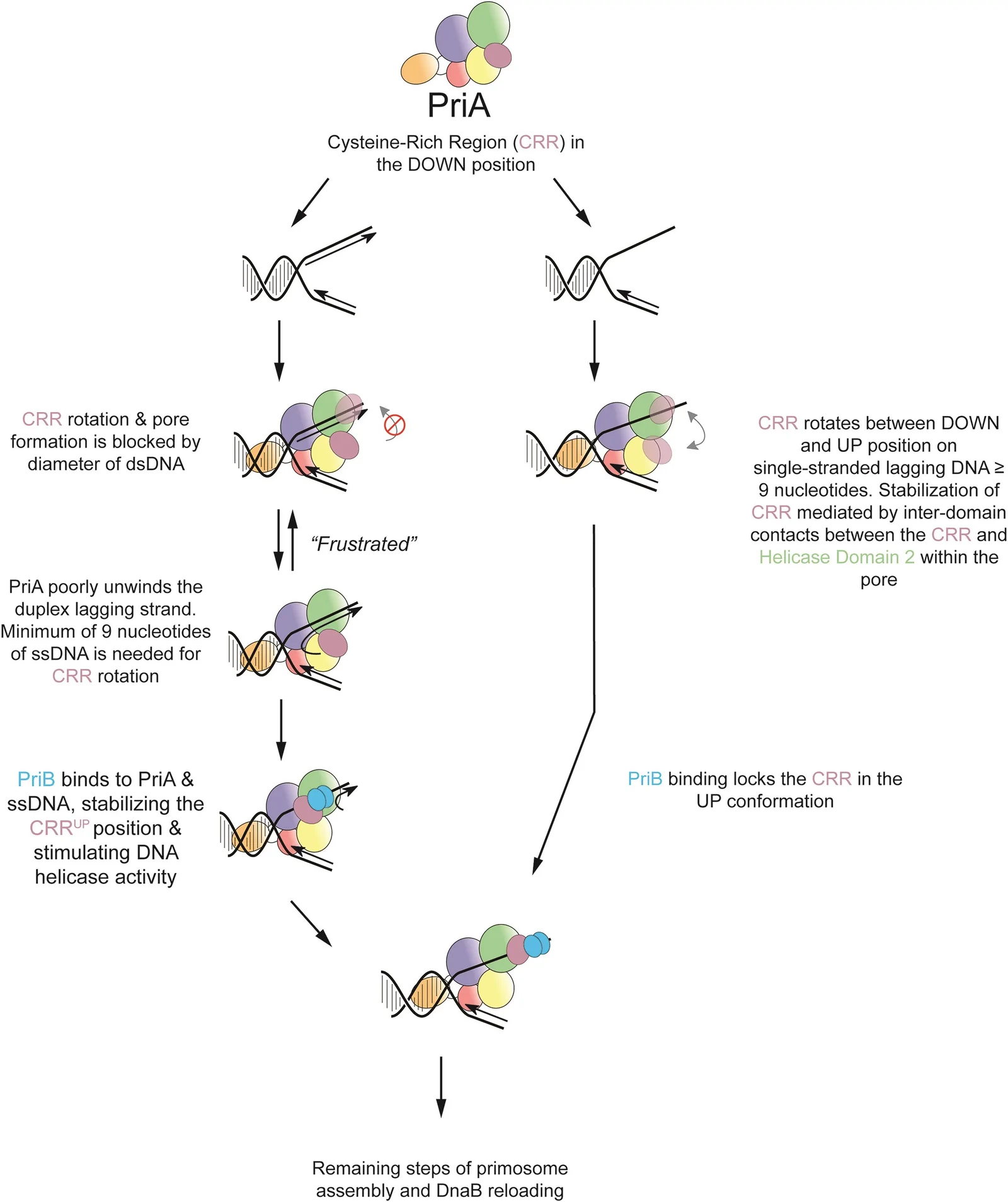
Replication fork structure influences the rotation and position of the PriA^CRR^. In the absence of DNA, PriA^CRR^ resides in the CRR^DOWN^ conformation. (Left) PriA binds to DNA replication forks with a dsDNA lagging strand primarily in the CRR^DOWN^ conformation. Formation of the CRR^UP^ conformation is prevented by the width of the dsDNA lagging strand. PriA in the CRR^DOWN^ conformation unwinds the lagging strand with poor processivity, allowing reannealing and resulting in frustrated DNA unwinding. PriB stimulates PriA helicase activity by stabilizing the CRR^UP^ conformation and binding ssDNA, which prevents reannealing of partially unwound DNA. (Right) PriA^CRR^ forms the CRR^UP^ conformation when ssDNA gap sizes of nine or more bases are present on the lagging strand. The CRR^UP^ conformation is stabilized by interdomain contacts between helicase domain 2 and the PriA^CRR^. PriB binding stabilizes the CRR^UP^ conformation with 11 or more ssDNA bases, prompting the next steps of preprimosome assembly and replication restart.

PriB strongly stimulated PriA helicase activity on replication forks with very short ssDNA lagging-strand gaps but had no measurable impact on forks with ssDNA gaps of nine or more bases (Fig. 2). However, PriB formed stable complexes with PriA only when PriA was bound to DNA substrates with longer ssDNA gaps. Moreover, mutations that alter the PriB docking site on the PriA^CRR^ or that reduce PriB ssDNA binding severely restrict PriB stimulation of PriA DNA unwinding [21, 26]. How then does PriB stimulate PriA helicase activity on replication forks with dsDNA lagging strands? One model is that PriB relieves “frustrated” DNA unwinding by PriA. Frustrated unwinding, in which a helicase repetitively unwinds dsDNA only to have the DNA reanneal, has been observed with other helicases [62, 63]. In such a model, PriA would unwind short stretches of lagging-strand dsDNA and then release the unwound products. If unwinding is insufficiently processive to fully separate the two DNA strands, the partially unwound products will reanneal, and PriA would be forced to reinitiate unwinding in a futile cycle (Fig. 6). For PriA, our data suggest that enhanced PriA^CRR^ dynamics would accompany lagging-strand unwinding once nine or more bases of ssDNA were exposed, giving PriA the opportunity to trap the parental ssDNA strand in the pore formed in the CRR^UP^ conformation. However, without PriB, the PriA^CRR^ position is highly dynamic when bound to a ssDNA lagging-strand structure, which could allow reannealing as the domain fluctuates between the CRR^UP^ and CRR^DOWN^ positions, resulting in frustrated unwinding. When PriB is included, its binding to PriA in the CRR^UP^ conformation would stabilize the conformation, allowing PriA to stably trap the ssDNA lagging-strand product and thus enhance unwinding. SSB, which can also stimulate PriA unwinding of similar replication forks to those tested here in a manner that is additive with PriB [23, 26], could also help to relieve frustrated DNA unwinding by binding to the displaced nascent lagging strand. Future single-molecule experiments similar to those performed in other cases [62, 63] could resolve whether PriA acts as a frustrated helicase and how PriB and/or SSB influence the unwinding reaction.

PriA variants designed to destabilize the CRR^UP^ conformation or ssDNA binding by helicase motif IV within the PriA pore have substantial impacts on preprimosome assembly, DNA helicase activity, ATP hydrolysis, and replication restart *in vivo*. Sequence changes to CRR^UP^ domain interface residues (H455 or E492), or motif IV ssDNA-binding residues (R427 or R428), severely impaired PriB binding, which is likely due to destabilization of the CRR^UP^ conformation in the variants (Fig. 3). The variants also displayed markedly reduced DNA helicase activity, which was only weakly enhanced by the addition of PriB in a subset of variants. While weakened helicase activity was expected for the helicase motif variants, it was not known whether the CRR^UP^ interdomain interface variants would be impaired as well. Taken together with experiments examining the impact of ssDNA gap lengths on PriA activity, these results suggest that the CRR^UP^ conformation of PriA is highly active as a helicase, with lower levels of activity in the CRR^DOWN^ conformation.

The interface variants also demonstrated an unexpected impact on PriA ATP hydrolysis. Variants designed to destabilize the CRR^UP^ conformation or ssDNA residues resulted in varied effects on ssDNA-stimulated ATP hydrolysis, ranging from severe reductions in activity for the motif IV changes to little effect on activity for the remaining changes (Fig. 5). In contrast, PriA variants designed to destabilize the CRR^DOWN^ interface significantly increased the ATP hydrolysis rate, even in the absence of DNA. These observations suggest the PriA^CRR^ position allosterically influences the ATPase active site, with the CRR^UP^ conformation displaying the highest ATPase levels. The modest resolution of the PriA/replication fork structures prevented detailed molecular analysis of potential changes in the ATPase active site in the CRR^UP^ conformation.

Finally, our investigation of the PriA variants in *E. coli* has expanded our understanding of the roles of PriA^CRR^  *in vivo*. Previous studies have shown that PriA ATPase and DNA helicase activities are not necessary for preprimosome assembly *in vitro* [15, 50]. PriA ATPase/helicase-deficient variants also support replication restart *in vivo* through the PriA/PriB pathway but not the PriA/PriC pathway [12, 21, 31, 50, 59]. We found that incorporation of the motif IV mutations into *priA* in *E. coli* significantly affected replication restart within the PriA/PriC pathway (i.e. in the absence of PriB) but not the PriA/PriB pathway, consistent with prior results. One outlier was the PriA H455A non-helicase motif CRR^UP^-destabilizing variant, which had very weak helicase activity *in vitro* and impaired both the PriA/PriB and PriA/PriC pathways when introduced into cells. Its behavior is consistent with the importance of the PriA^CRR^ in both helicase function and PriB docking.

Taken together, our results link PriA conformational flexibility to its ability to accommodate diverse DNA replication fork structures and suggest that the position of the PriA^CRR^ strongly influences DNA unwinding and ATP hydrolysis and must be sufficiently stabilized by interdomain contacts to mediate preprimosome assembly. These results support a model in which PriA dynamics serve as a conformational switch regulating preprimosome formation, ensuring that replication restart is restricted to bona fide replication fork structures that have been prepared for replicative helicase loading onto the ssDNA lagging strand.

## Supplementary Material

gkag911_Supplemental_Files

## Data Availability

Cryo-EM maps have been deposited in the Electron Microscopy Data Bank (EMDB) with accession codes (EMD-76387, EMD-76388, and EMD-76389). All original biochemical data will be freely available through the Dryad repository at https://doi.org/10.5061/dryad.wpzgmsc40. We thank the University of Wisconsin-Madison Biophysics Instrumentation Facility for its assistance in collecting and analyzing Mass Photometry data.
