# Supplementary material for: Domain dynamics within the PriA DNA helicase regulate DNA replication restart
Source: Nucleic Acids Res. 2026 Sep 22;54(18):gkag911. doi: 10.1093/nar/gkag911 (PMC13594977; doi:10.1093/nar/gkag911)
Supplement: Supplementary file 1 [file gkag911_supplemental_files.zip › Deorio et al, revised SI.pdf]

SUPPLEMENTARY INFORMATION CONTENT

Supplementary Tables S1-S5

Supplementary Figures S1-S13

Supplementary Figure and Movie S1 legends

Supplementary References

Supplementary Table S1: Oligonucleotides used to construct synthetic replication forks.

Oligo name	Sequence (5′ - 3′)	Description	Substrate name	Reference
oAD027	CGA GAC CGC AAT ACG GAT AAG GGC TGA GCA CGC CGA CGA A	3-Stranded EM fork Leading template strand	Fork 1 & 2	(1)
oAD028	GCC GCA GAC TCA TTT AGC CCT TAT CCG TAT TGC GGT CTC G	3-Stranded EM fork Lagging template strand	Fork 1 & 2	(1)
oAD029	TTC GTC GGC GTG CTC	3-Stranded EM fork Leading nascent strand	Fork 1 & 2	(1)
oAD038	AGT CTG CGG C	4-Stranded EM fork Lagging nascent strand	Fork 2	This work
1b-98	GCA AGC CTT CTA CAG GTC GAC CGT CCA TGG CGA CTC GAG ACC GCA ATA CGG ATA AGG GCT GAG CAC GCC GAC GAA CAT TCA CCA CGC CAG ACC ACG TA	Leading parental strand for radiolabeled EMSA and helicase assays	Fork 3, 4, 5, 6, 7, 8	(2)
3L-98	GAC TAT CTA CGT CCG AGG CTC GCG CCG CAG ACT CAT TTA GCC CTT ATC CGT ATT GCG GTC TCG AGT CGC CAT GGA CGG TCG ACC TGT AGA AGG CTT GC	Lagging parental strand for radiolabeled EMSA and helicase assay	Fork 3, 4, 5, 6, 7, 8	(2)
11b-38	TAC GTG GTC TGG CGT GGT GAA TGT TCG TCG GCG TGC TC	Leading nascent strand for radiolabeled EMSA and helicase assay	Fork 3, 4, 5, 6, 7, 8, 16, 17	(2)
b-33	AGT CTG CGG CGC GAG CCT CGG ACG TAG ATA GTC	Lagging nascent strand for radiolabeled EMSA and helicase assay with 5 nt gap	Fork 3, 16, 17	(2)
oTW109_9nt gap	TGC GGC GCG AGC CTC GGA CGT AGA TAG TC	Lagging nascent strand for radiolabeled EMSA and helicase assay with 9 nt gap	Fork 5	This work
oTW109_10 ntgap	GC GGC GCG AGC CTC GGA CGT AGA TAG TC	Lagging nascent strand for	Fork 6	This work

		radiolabeled EMSA and helicase assay with 10 nt gap		
oTW109_11 ntgap	CGG CGC GAG CCT CGG ACG TAG ATA GTC	Lagging nascent strand for radiolabeled EMSA and helicase assay with 11 nt gap	Fork 7	This work
oTW109_15 ntgap	GCG AGC CTC GGA CGT AGA TAG TC	Lagging nascent strand for radiolabeled EMSA and helicase assay with 15 nt gap	Fork 8	This work
oHD162	ACG TCC GAG GCT CGC GCC GCA GAC TCA TTT AGC CCT TAT CCG TAT TGC GGT CTC G	Lagging template for Mass Photometry	Fork 9, 10, 11, 12, 13, 14, 15	This work
oHD153	CGA GAC CGC AAT ACG GAT AAG GGC TGA GCA CGC CGA CGA ACA TTC ACC ACG CCA C	Leading template for Mass Photometry	Fork 9, 10, 11, 12, 13, 14, 15	This work
oHD117	GTG GCG TGG TGA ATG TTC GTC GGC GTG CTC	Leading nascent for Mass Photometry	Fork 9, 10, 11, 12, 13, 14, 15	This work
oHD156	AGT CTG CGG CGC GAG CCT CGG ACG T	Lagging nascent strand for Mass Photometry 5 nt gap	Fork 10	This work
oHD157	TCT GCG GCG CGA GCC TCG GAC GT	Lagging nascent strand for Mass Photometry 7 nt gap	Fork 11	This work
oHD158	TGC GGC GCG AGC CTC GGA CGT	Lagging nascent strand for Mass Photometry 9 nt gap	Fork 12	This work
oHD159	CGG CGC GAG CCT CGG ACG T	Lagging nascent strand for Mass Photometry 11 nt gap	Fork 13	This work
oHD160	GCG CGA GCC TCG GAC GT	Lagging nascent strand for Mass Photometry 13 nt gap	Fork 14	This work
oHD161	GCG AGC CTC GGA CGT	Lagging nascent strand for Mass Photometry 15 nt gap	Fork 15	This work

oHD190	GAC TAT CTA CGT CCG AGG CTC GCG CCG CAG ACT CAT TTA GCC CTT ATC CGT ATT GCG GTC TCG AGT CGCCAT GGA CGG	Lagging template strand for Helicase assay 5 nt gap and 30 nt duplex lagging	Fork 16	This work
oHD191	CCG TCC ATG GCG ACT CGA GAC CGC AAT ACG GAT AAG GGC TGA GCA CGC CGA CGA ACA TTC ACC ACGCCA GAC CAC GTA	Leading template strand for Helicase assay with 30 nt duplex lagging	Fork 16, 17	This work
oHD192	GAC TAT CTA CGT CCG AGG CTC GCG CCG CAG ACT GTA CAT TAG TCA TTT AGC CCT TAT CCG TAT TGC GGTCTC GAG TCG CCA TGG ACG G	Lagging template strand for Helicase assay 15 nt gap and 30 nt duplex lagging	Fork 17	This work

Supplemental Table S2: Strain list for construction of PriA^{CRR} variants

Strain #	*priA*	*priB*	*priC*	Other Relevant Genotype	Reference or Derivation
CAG5052	+	+	+	*btuB3191::Tn10 metB1*	(3)
JC19165	+	+	*303::kan*		(4)
JC19295	*2::kan*	+	+	*zjj-202::Tn10 dnaC809,820*	(5)
SS1092	+	+	+	*dnaC809,820*	(4)
SS1100	+	+	*303::kan*	*dnaC809,820*	JC19165 -> SS1092 [b]
SS1441	*priA300*	+	+		(5)
SS4278	+	+	+	*del(galK)100::kan*	(6) Keio Collection
SS6282	+	+	+	*hupA::mcherry::kan*	(7)
SS6321 [a]	+	+	+		(7)
SS6796	+	*del(302)*	+	*zjf-599::Tn10*	(7)
SS7064	*2::kan*	+	+		JC19295 -> SS6321 [b]
SS8974	+	+	+	*btuB3191::Tn10 metB1*	CAG5052 -> SS6321 [c]
SS9114	+	+	*303::kan*		SS1100 -> SS6321 [b]
SS9116	+	*del(302)*	+	*zjf-599::Tn10*	SS6796 ->SS6321 [c]
SS9141	+	+	*303::kan*	*btuB3191::Tn10 metB1*	CAG5052 -> SS9114 [c]
SS10029	+	+	+	*dnaC809,820 del(galK)200::kan*	SS4278 -> SS1092 [b]
SS10034	+	+	+	*dnaC809,820 del(galK)200::frt*	SS10029 flip KanS with pCP20
SS11301	+	+	+	*dnaC809,820*	pKD46/SS1092
SS11366	+	+	+	*btuB3191::Tn10 metB1 dnaC809,820*	CAG5052 -> SS1092 [c]
SS13526	+	+	+	*del(metB)100::kan*	(6)- Keio Collection

SS13598	*355 (D438A, T482A, H483A)::cat*	+	+		(1)
SS14050	*300 (K230R)::cat*	+	+		This work
SS14080	*del(priA)358::kan*	+	+	*dnaC809,820*	This work
SS14086	*del(priA)358::kan*	+	+	*dnaC809,820*	SS14080 -> SS11366 [b]
SS14207	+	+	+	*del(metB)100::kan*	SS13526 -> SS9116 [b]
SS14362	+	+	+	*del(metB)100::kan dnaC809,820 del(galK)200::frt*	SS13526 -> SS10034 [b]
SS14446	*del(priA)367::kan*	+	+	*dnaC809,820 del(galK)200::frt*	This work
SS14453	*del(priA)367::kan*	+	+	*dnaC809,820 del(galK)200::frt*	SS14446 -> SS14362 [b]
SS14462	*374 (R427A) ::cat*	+	+	*dnaC809,820 del(galK)200::frt*	This work
SS14463	*375 (R427D) ::cat*	+	+	*dnaC809,820 del(galK)200::frt*	This work
SS14464	*376 (R428A) ::cat*	+	+	*dnaC809,820 del(galK)200::frt*	This work
SS14465	*377 (R428D) ::cat*	+	+	*dnaC809,820 del(galK)200::frt*	This work
SS14467	*374 (R427A) ::cat*	+	+		SS14462 -> SS8974 [d]
SS14468	*375 (R427D) ::cat*	+	+		SS14463 -> SS8974 [d]
SS14469	*376 (R428A) ::cat*	+	+		SS14464 -> SS8974 [d]
SS14470	*377 (R428D) ::cat*	+	+		SS14465 -> SS8974 [d]
SS14477	*374 (R427A) ::cat*	+	*303::kan*		SS14462 -> SS9141 [d]
SS14478	*376 (R428A) ::cat*	+	*303::kan*		SS14464 -> SS9141 [d]
SS14480	*374 (R427A) ::cat*	*del(302)*	+	*zjf-599::Tn10*	SS14462 -> SS14207 [d]
SS14481	*376 (R428A) ::cat*	*del(302)*	+	*zjf-599::Tn10*	SS14464 -> SS14207 [d]
SS14483	*378 (H437A) ::cat*	+	+	*dnaC809,820 del(galK)200::frt*	This work
SS14484	*379 (H455A) ::cat*	+	+	*dnaC809,820 del(galK)200::frt*	This work

SS14485	*378 (H437A) ::cat*	+	+		SS14483 -> SS8974 [d]
SS14486	*379 (H455A) ::cat*	+	+		SS14484 -> SS8974 [d]
SS14487	*378 (H437A) ::cat*	+	*303::kan*		SS14483 -> SS9141 [d]
SS14488	*378 (H437A) ::cat*	*del(302)*	+	*zjf-599::Tn10*	SS14483 -> SS14207 [d]
SS14489	*379 (H455A) ::cat*	+	*303::kan*		SS14484 -> SS9141 [d]
SS14490	*379 (H455A) ::cat*	*del(302)*	+	*zjf-599::Tn10*	SS14484 -> SS14207 [d]
SS14493	*380 (H600A) ::cat*	+	+	*dnaC809,820 del(galK)200::frt*	This work
SS14494	*380 (H600A) ::cat*	+	+		SS14493 -> SS8974 [d]
SS14495	*380 (H600A) ::cat*	+	*303::kan*		SS14493 -> SS9141 [d]
SS14496	*380 (H600A) ::cat*	*del(302)*	+	*zjf-599::Tn10*	SS14493 -> SS14207 [d]

[a] The complete genotype of SS6313 is *hupA-mcher (translational fusion) sulAp-gfp (transcriptional fusion) sulB103 del(lacMS286) attphi80::phi80::del(lacBK1) argE3 his-4 thi-1pro rpsL31 xyl-5 mtl-1 tsx.*

[b] Select for Kan^R and then screen for the relevant mutation or phenotype.

[c] Select for Tet^R and then screen for the relevant mutation or phenotype.

[d] Select for growth on minimal media without methionine and then screen for the relevant mutation or phenotype.

Supplemental Table S3: Notes on construction of *priA* alleles on chromosome

***priA* allele**	**Notes on Constructions [a]**
priA300 (K230R)::cat	Linear DNA generated from PCR with prSJS1665/1237 on SS13598 was used to transform pKD46/SS1441. Select for CatR.
priA374 (R427A)::cat	Linear DNA generated from PCR with prSJS1774/1237 on DNA generated by PCR with prSJS1770/1237 on SS14050 was then used to transform pKD46/SS14453. Select for CatR and screen KanS AmpS.
priA375 (R427D)::cat	Linear DNA generated from PCR of prSJS1774/1237 on DNA generated by PCR with prSJS1771/1237 on SS14050 was used to transform pKD46/SS14453. Select for CatR and screen KanS AmpS.
priA376 (R428A)::cat	Linear DNA generated from PCR of prSJS1774/1237 on DNA generated by PCR with prSJS1772/1237 on SS14050 was used to transform pKD46/SS14453. Select for CatR and screen KanS AmpS.
priA377 (R428D)::cat	Linear DNA generated from PCR of prSJS1774/1237 on DNA generated from PCR with prSJS1773/1237 on SS14050 was used to transform pKD46/SS14453. Select for CatR and screen KanS AmpS.
priA378 (H437A)::cat	Linear DNA generated from PCR of prSJS1769/1237 on pPriA378 and then treated with DpnI was used to transform pKD46/SS14453. Select CatR and screen KanS and AmpS.
priA379 (H455A)::cat	Linear DNA generated from PCR of prSJS1769/1237 on pPriA379 and then treated with Dpn I was used to transformed pKD46/SS14453. Select CatR and screen KanS and AmpS.
priA380 (H600A)::cat	Linear DNA generated from PCR of pPriA380 with prSJS1235/1237 was used to transform pKD46/SS14086.
del(priA)358::kan	Linear DNA generated from PCR of prSJS1728/1266 on SS6282 was used to transform pKD46/SS11301. Select for KanR.
del(priA)367::kan	Linear DNA generated from PCR of prSJS1751/1266 on SS6282 was used to transform pKD46/SS10034. Select KanR.

[a] After placing the new alleles on the chromosome, all constructions were confirmed by DNA sequencing. Only the sequences of the *priA* gene that had been amplified by PCR and used for recombination were verified.

Supplementary Table S4: Primer list for construction of PriA^{CRR} variants

Name	Sequence 5′ to 3′	Description
prSJS1237	CCTGGCACGTCAGGCGTACTACATCC	Reverse primer just after *priA* gene
prSJS1266	ACAGAATTTTCATGAAAAGTGTGATGAATATTGAATTTTTCGATCCGCCTCGCATCGTGAGCGGTCGCAATCTGTAGGCTGGAGCTGC	Reverse primer just after *priA* gene and *kan* gene in in *hupA-mcher-kan* (SS6282)
prSJS1665	CGAGAAACTGTGGGTTCTCGGTCCGGTTCCGGCTCTGGCACC	Forward primer including the single Rsr II site of *priA* gene
prSJS1728	GGCAGAAGTACATCGCGGCGGCGCGCGGATTTTGATTGGTACACAAATGCTGGGGATCCGTCGACCTGCAGTTCG	Forward primer with homology to codons 525-543 of *priA* and the *kan* gene in *hupA-mcher-kan* (SS6282)
prSJS1751	GCGTTAATCACTCGTATGCGCCAGCATTTACAGGCTGATAACCAGGTCATTTGAGGATCCGTCGACCTGCAGTTCG	Forward primer to make deletion of *priA* starting at codon 423
prSJS1769	GGTCAGAAGGTGCAGGCAGGTCTGGCTCC	Forward primer in *priA* near codon 405
prSJS1770	CGTATGCGCCAGCATTTACAGGCTGATAACCAGGTCATTCTCTTTCTTAACGCCCGTGGCTTTGCGCCTGCACTGC	Forward primer creating mutation *priA374* (R427A)
prSJS1771	CGTATGCGCCAGCATTTACAGGCTGATAACCAGGTCATTCTCTTTCTTAACGACCGTGGCTTTGCGCCTGCACTGC	Forward primer creating mutation *priA375* (R427D)
prSJS1772	CGTATGCGCCAGCATTTACAGGCTGATAACCAGGTCATTCTCTTTCTTAACCGCGCTGGCTTTGCGCCTGCACTGC	Forward primer creating mutation *priA376* (R428A)
prSJS1773	CGTATGCGCCAGCATTTACAGGCTGATAACCAGGTCATTCTCTTTCTTAACCGCGATGGCTTTGCGCCTGCACTGC	Forward primer creating mutation *priA377* (R428D)
prSJS1774	GGATTTAAAAGGTCAGAAGGTGCAGGCAGGTCTGGCTCCGGCGTTAATCACTCGTATGCGCCAGCATTTACAGGC	Forward primer in *priA*

Supplementary Table S5: *In vivo* characterization of CRR^{DOWN}-destabilized PriA variants.

**Strain**	***priA***	***priB***	***priC***	**Avg. Cell Length**	**Rel. SOS Intensity**	**Chromosome Partitioning**
SS6321	+	+	+	640	1.1	+
SS7064	*null*	+	+	1670	6.2	-
SS9116	+	*null*	+	710	2.1	+
SS9114	+	+	*null*	630	1.7	+
SS14486	H437A	+	+	630	1.9	+
SS14488	H437A	*null*	+	730	1.8	+
SS14487	H437A	+	*null*	720	1.9	+
SS14494	H600A	+	+	640	2.0	+
SS14496	H600A	*null*	+	820	2.5	-/+
SS14495	H600A	+	*null*	590	1.9	+

SUPPLEMENTARY FIGURES

Supplementary Figure S1

A.

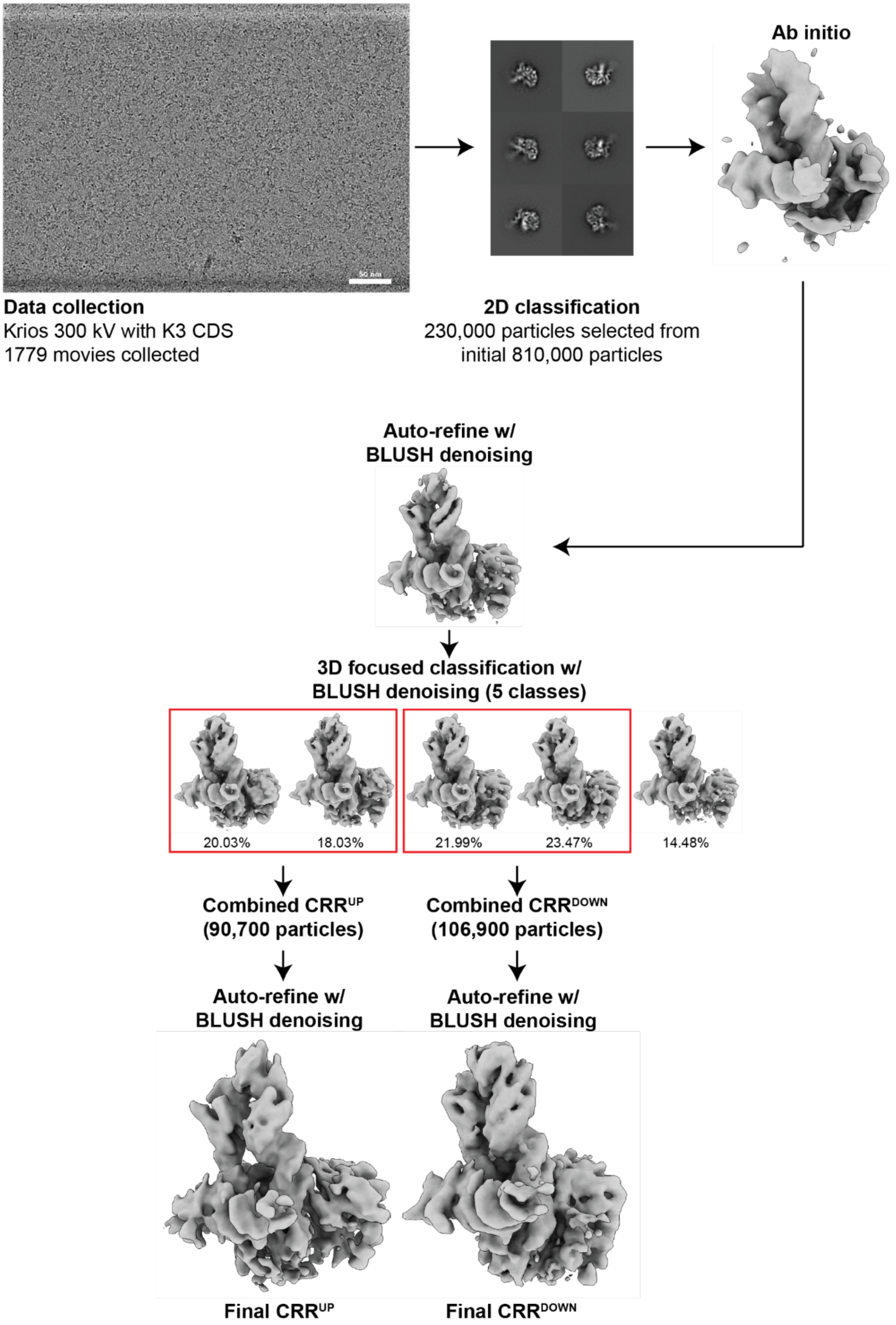

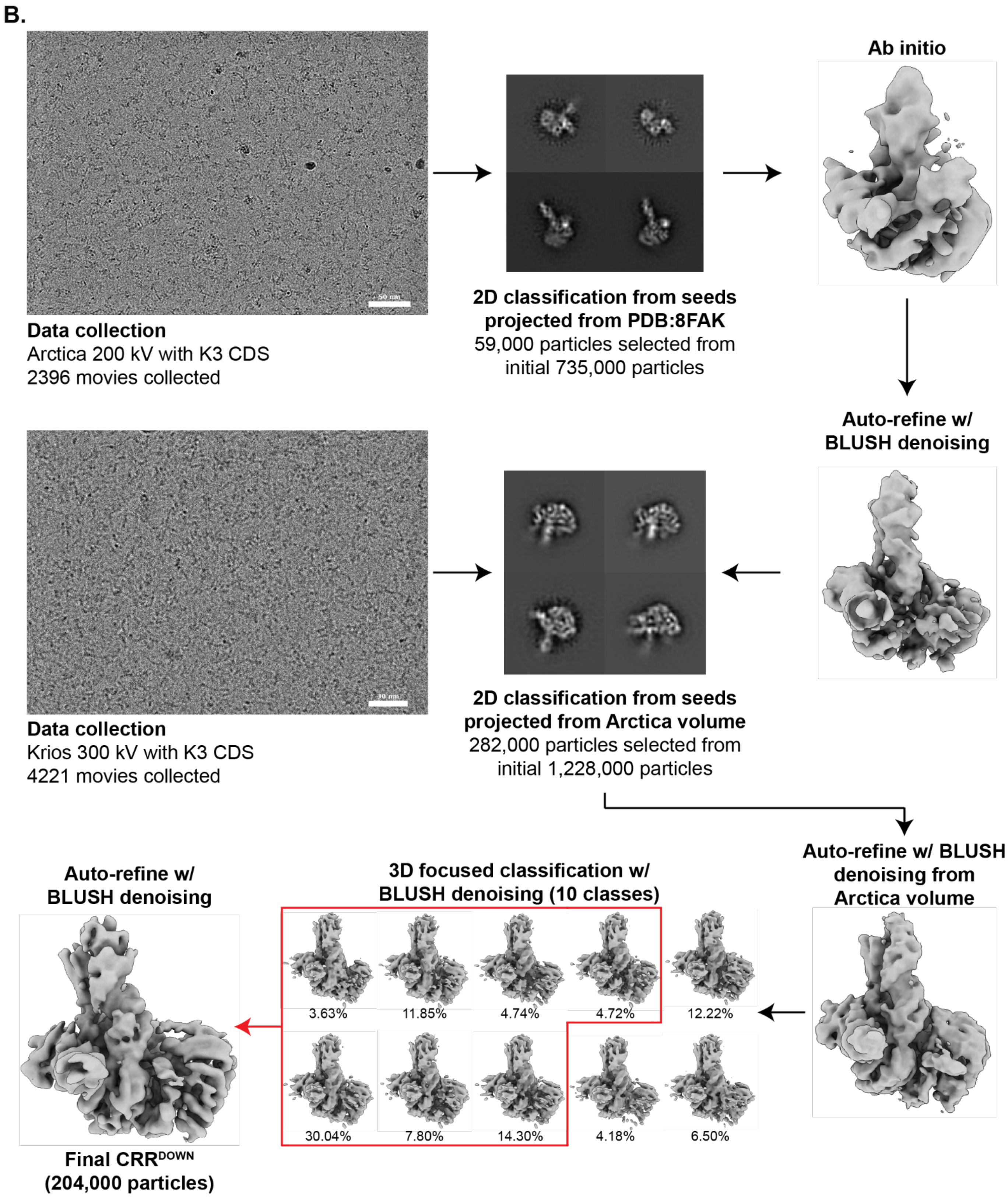

B.
Ab initio
Data collection
Arctica 200 kV with K3 CDS
2396 movies collected
2D classification from seeds
projected from PDB:8FAK
59,000 particles selected from
initial 735,000 particles
Auto-refine w/
BLUSH denoising
Data collection
Krios 300 kV with K3 CDS
4221 movies collected
2D classification from seeds
projected from Arctica volume
282,000 particles selected from
initial 1,228,000 particles
Auto-refine w/ BLUSH
denoising from
Arctica volume
3D focused classification w/
BLUSH denoising (10 classes)
3.63%
11.85%
4.74%
4.72%
12.22%
30.04%
7.80%
14.30%
4.18%
6.50%
Auto-refine w/
BLUSH denoising
Final CRR^DOWN
(204,000 particles)

Supplementary Figure S2

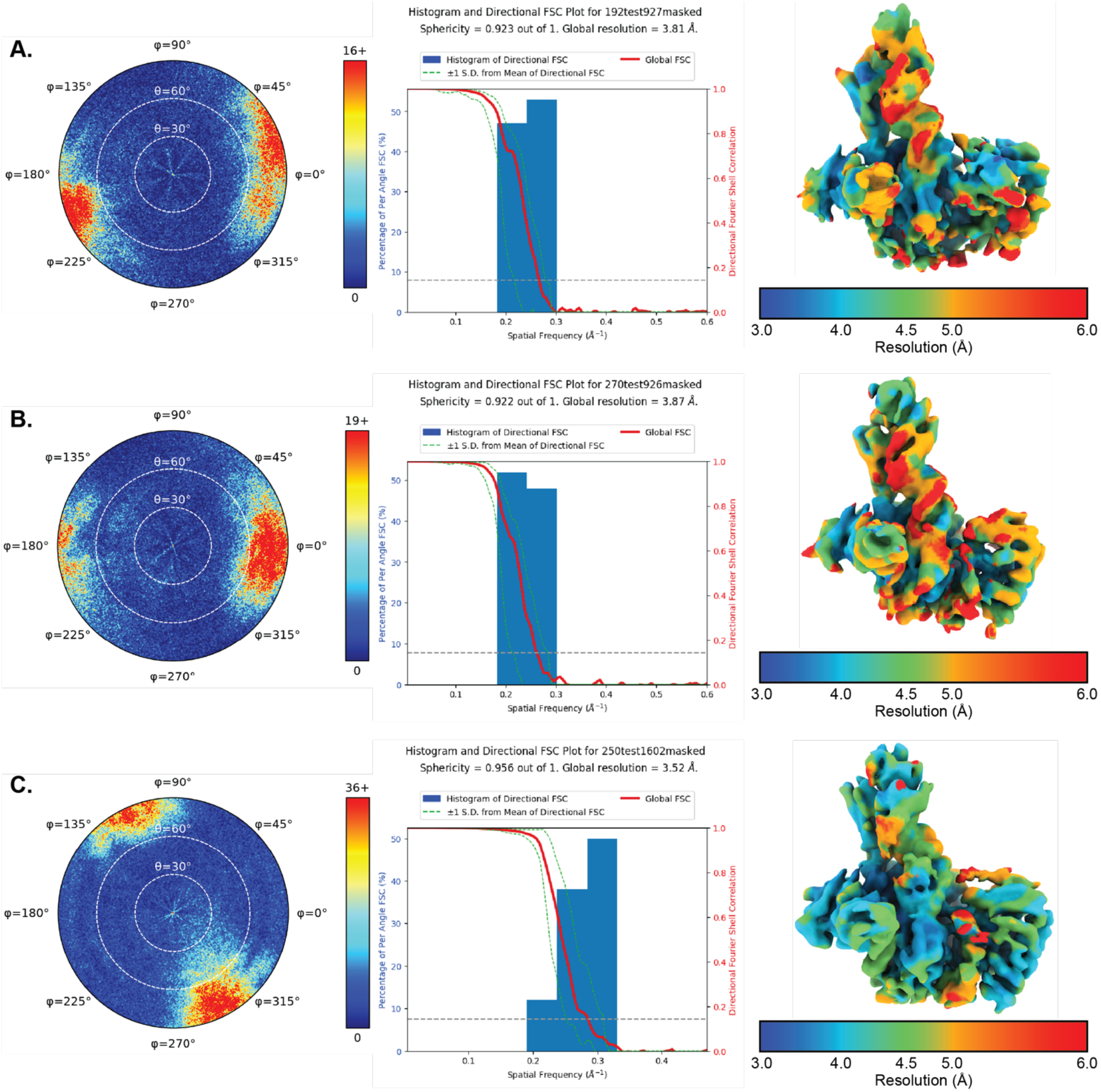

Supplementary Figure S3

A.

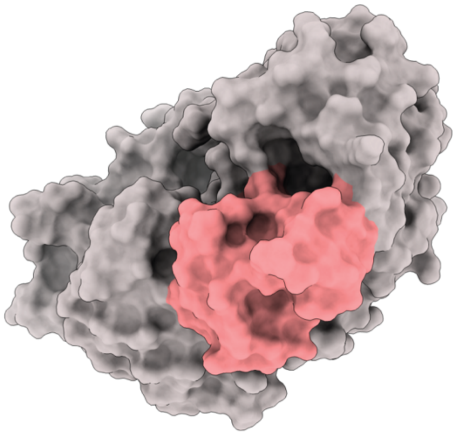

Apo PriA
PDB ID: 6DCR

B.

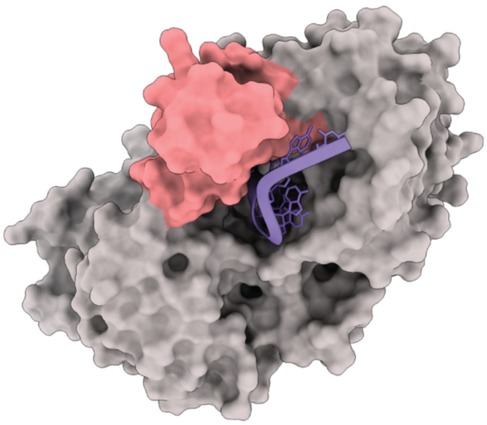

PriA/PriB/DNA replication fork
PDB ID: 8FAK

Supplementary Figure S4

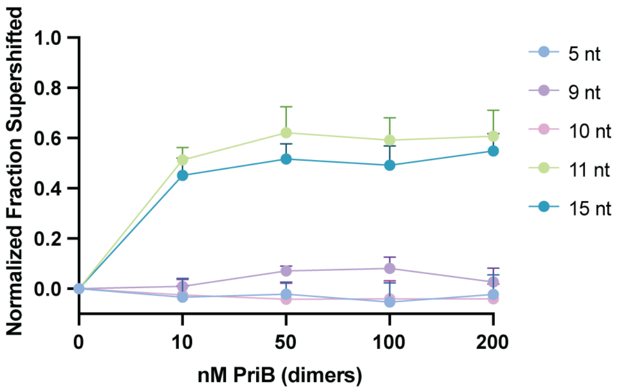

Supplementary Figure S5

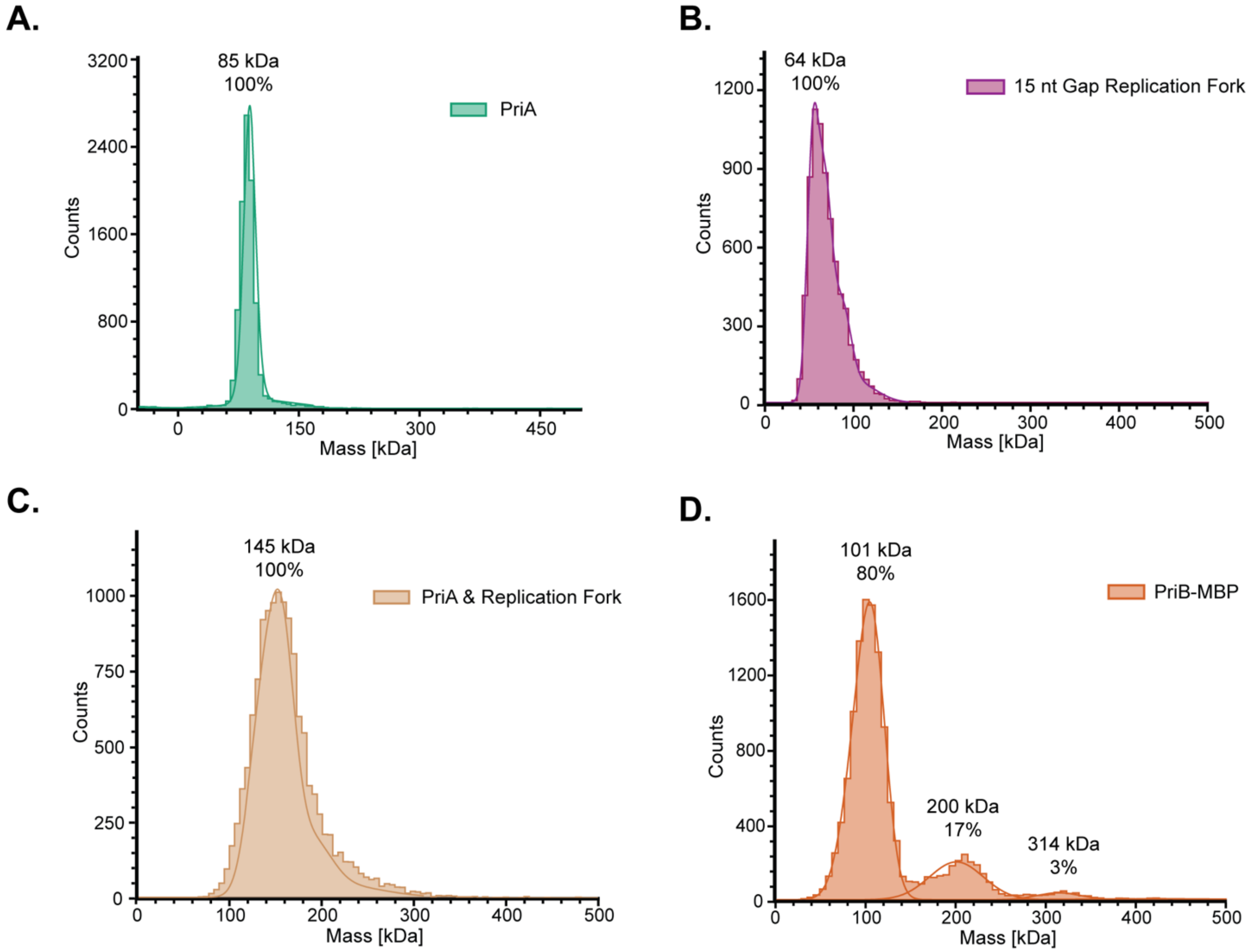

Supplementary Figure S6

A.

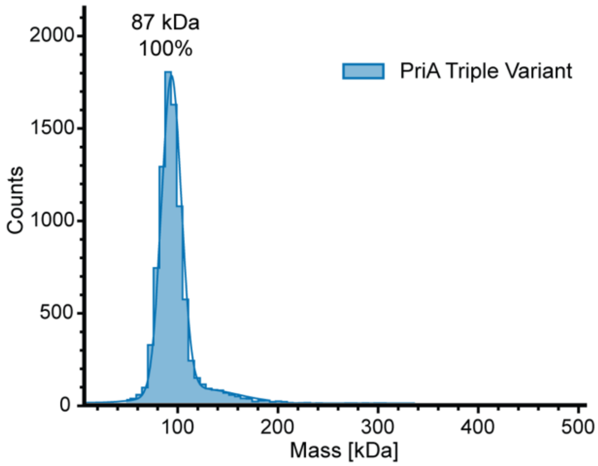

B.

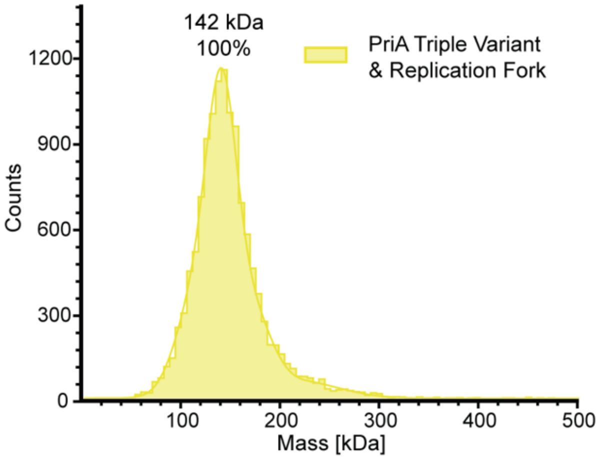

C.

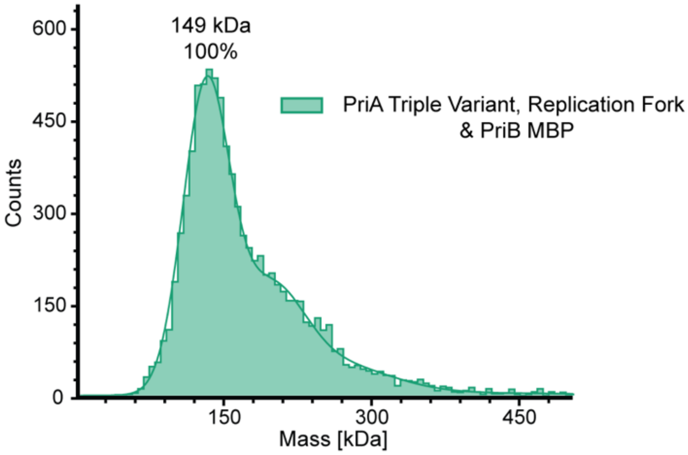

Supplementary Figure S7

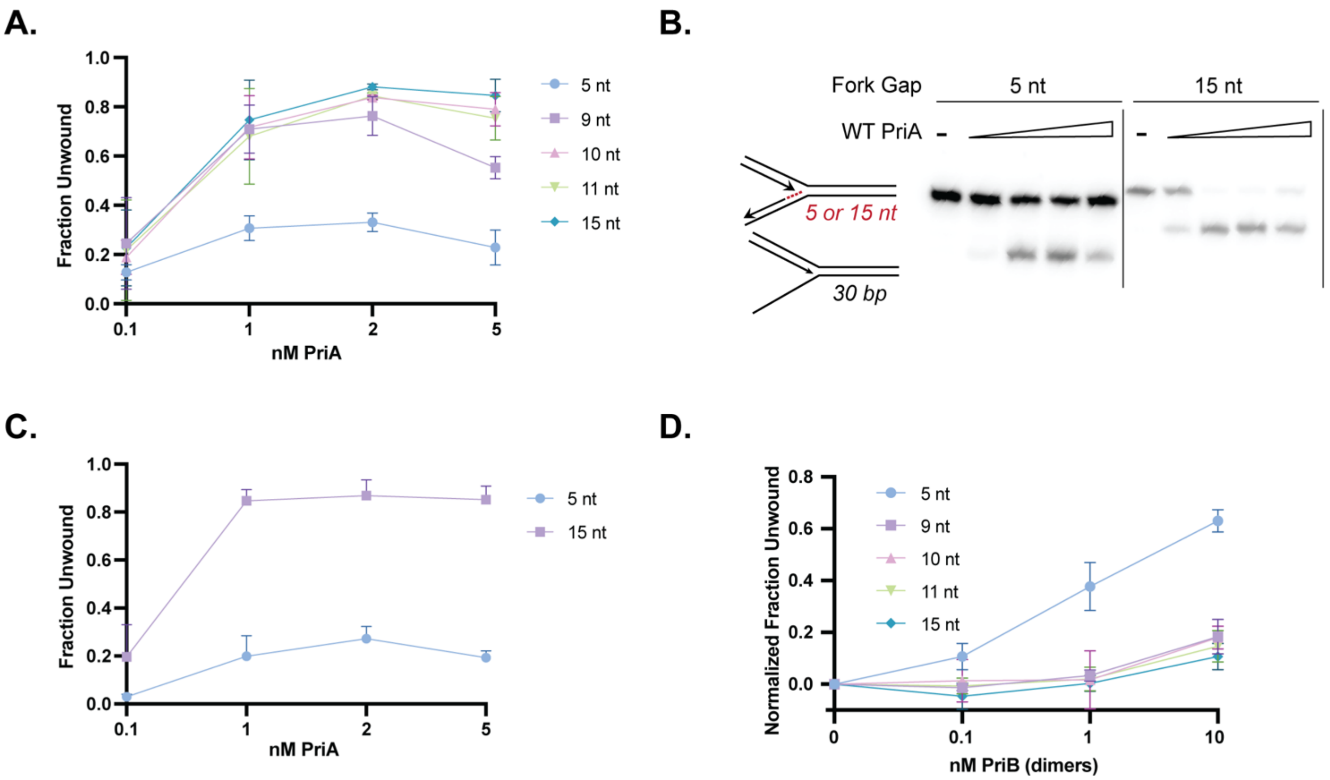

Supplementary Figure S8

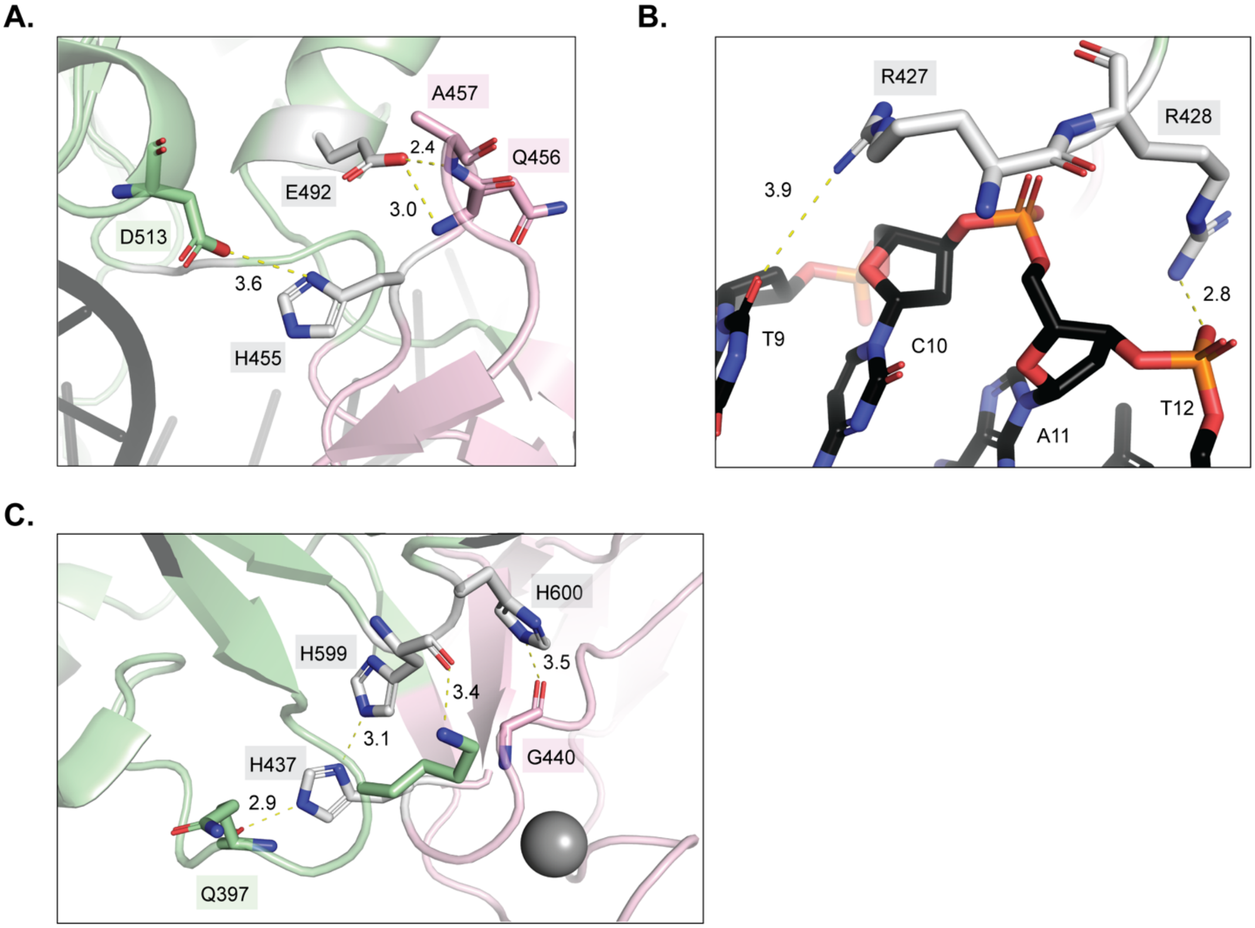

Supplementary Figure S9

A.

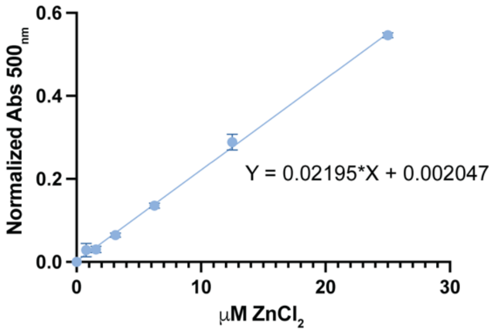

B.

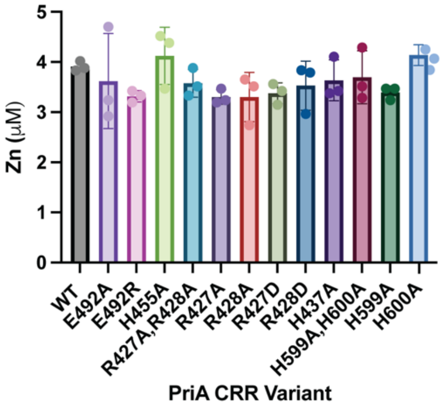

Supplementary Figure S10

A.

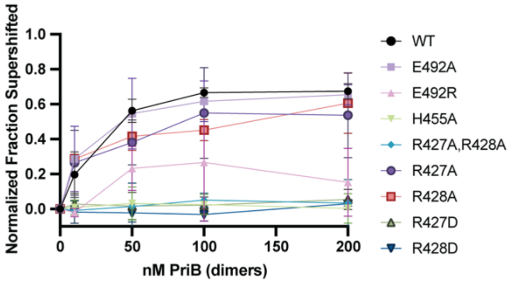

B.

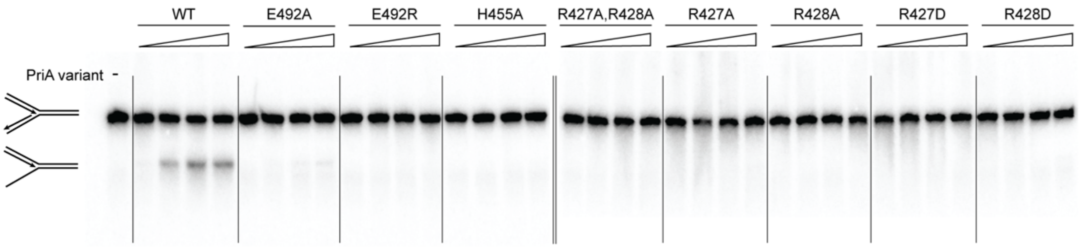

C.

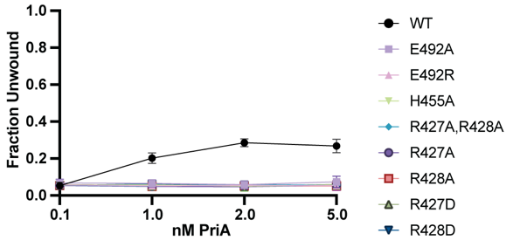

D.

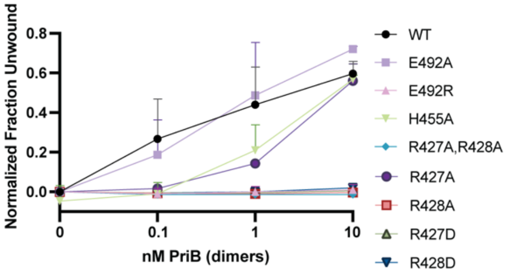

Supplementary Figure S11

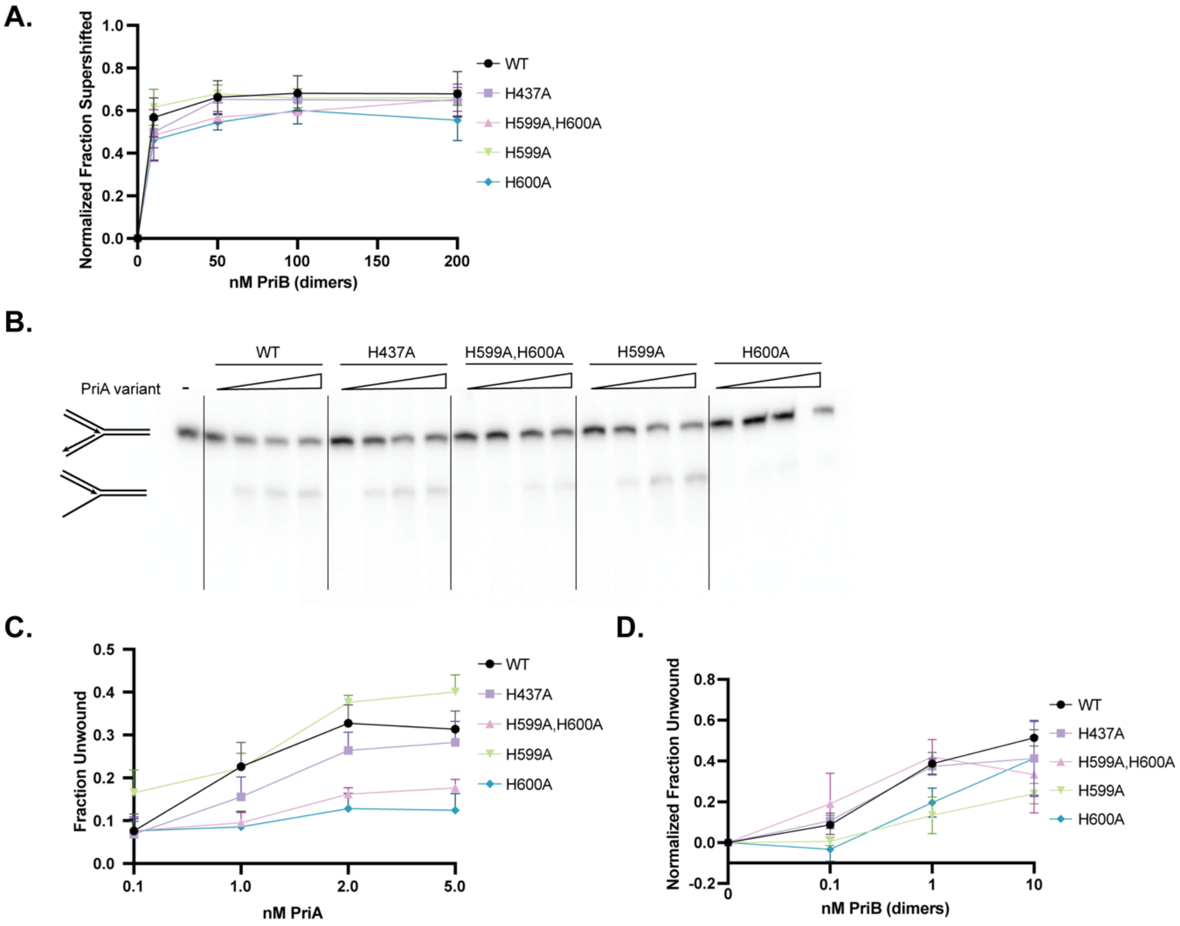

Supplementary Figure S12

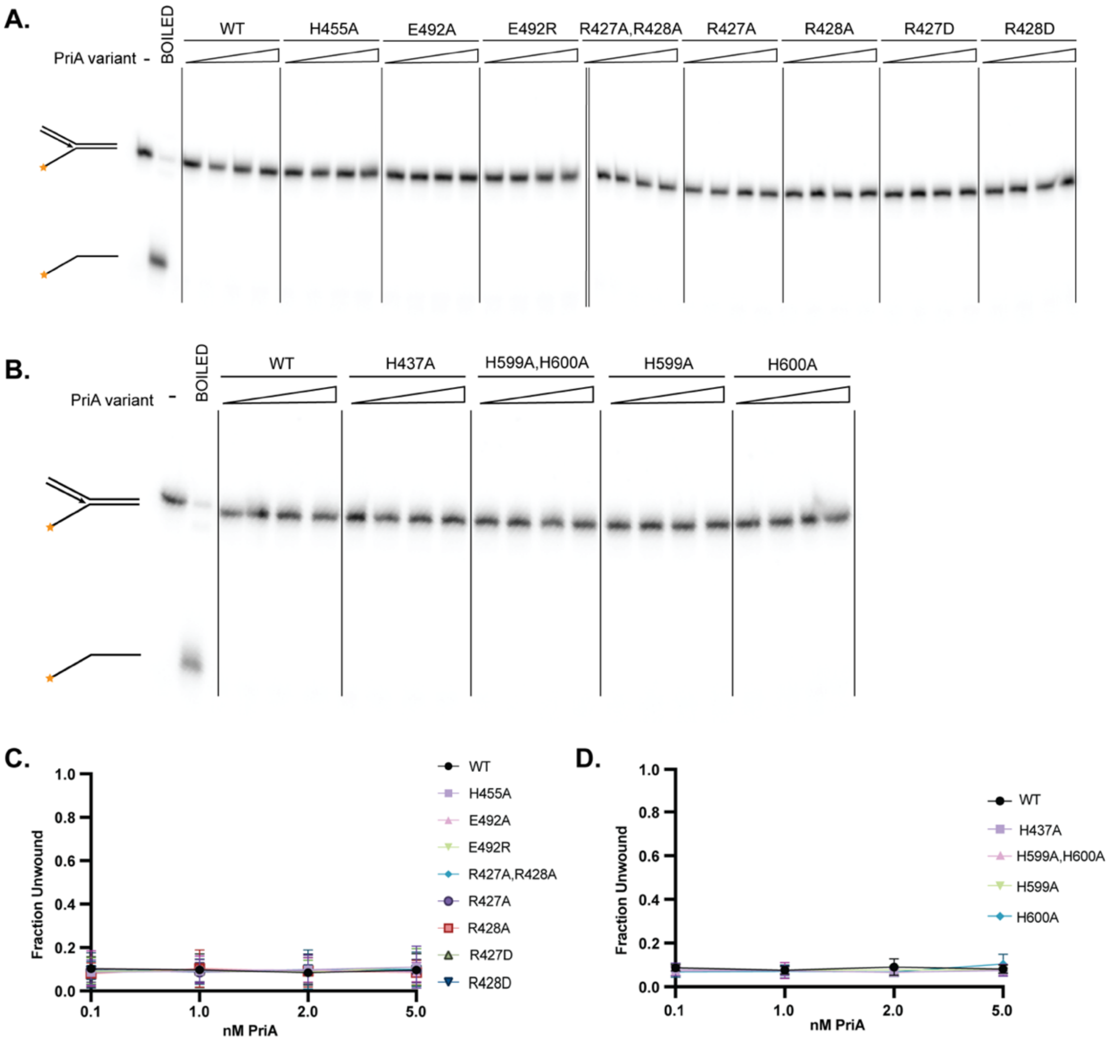

Supplementary Figure S13.

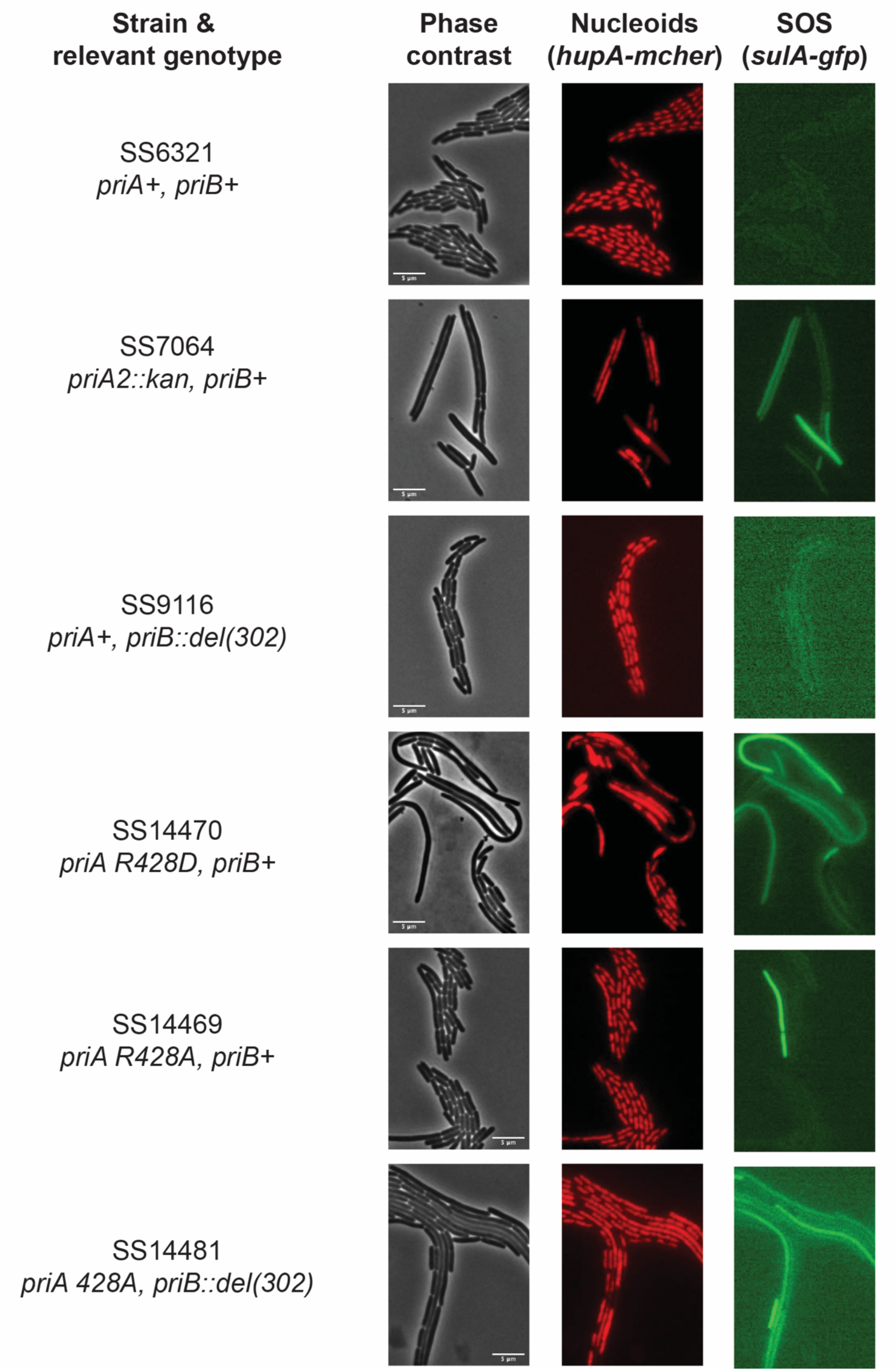

SUPPLEMENTARY FIGURE AND MOVIE LEGENDS

Supplementary Figure S1. Cryo-EM data processing workflow. Workflow for cryo-EM structure determination and resulting density maps for the PriA/3-stranded **(A)** and PriA/4-stranded **(B)** datasets. Representative cryo-EM micrographs from data collection and selected 2D class averages showing distinct particle views and conformations are shown. 3D reconstructions for all steps in the processing pipeline and particle counts are shown for the final cryo-EM densities.

Supplementary Figure S2. Angular distribution, 3D FSC plots, and local resolution maps for cryo-EM volumes. Angular distribution plots illustrating particle orientation coverage, 3D Fourier Shell Correlation (FSC) (8) plots that illustrate map quality and resolution estimates, and local resolution maps colored by resolution for the 3-stranded fork CRR^{UP} **(A)**, 3-stranded fork CRR^{DOWN} **(B)**, and 4-stranded fork CRR^{DOWN} **(C)** cryo-EM densities.

Supplementary Figure S3. DNA-dependent conformational change of the cysteine-rich region in PriA. A. Crystal structure of apo *E. coli* PriA (PDB ID: 6DCR)(7). $PriA^{CRR}$ (pink) resides in the CRR^{DOWN} position. **B.** Cryo-EM structure of PriA in complex with a synthetic DNA replication fork (PDB ID: 8FAK, PriB omitted from image) (1). $PriA^{CRR}$ (pink) rotates ~80° to the CRR^{UP} position, generating a pore encircling the lagging template strand (purple).

Supplementary Figure S4. PriB binding to PriA-DNA complex requires 11-bases of lagging strand ssDNA. Quantification of the fraction of PriB-supershifted (PriA/PriB/replication fork complex) to PriA-shifted (PriA/replication fork) bands in Figure 2A. Normalized to 0 nM PriB in Figure 2A. Data points are the mean of at least three independent measurements, with error bars representing the standard deviation.

Supplementary Figure S5: Mass photometry molecular weight standards. A. Mass profile for WT PriA (~85 kDa). **B.** Mass profile for a representative DNA replication fork containing a 15-base ssDNA lagging strand (Fork 15) (~64 kDa). **C.** Mass profile for WT PriA bound to Fork 15 (~145 kDa). **D.** Mass profile for PriB-MBP fusion. PriB-MBP homodimer is ~101 kDa. Higher-molecular-weight species are observed with PriB-MBP.

Supplementary Figure S6: PriB requires the PriA^{CRR} interface for stable binding to the PriA/replication fork complex. A. Mass profile for PriA triple variant D438A, S481A, T482A (~87 kDa). **B.** Mass profile for PriA triple variant bound to Fork 9 (~142 kDa). **C.** Mass profile for PriA triple variant, Fork 9, and PriB MBP (~149 kDa).

Supplementary Figure S7: Apo PriA helicase activity. A. Quantification of fraction unwound of DNA replication forks containing ssDNA gaps (5-, 9-, 10-, 11-, 15-bases) between the fork junction and duplexed lagging strand, as depicted in Figure 2C. **B.** PriA unwinding of DNA replication forks containing ssDNA gaps (5 or 15bases) with 30 bp lagging strand. Forks 16 and 17 (1 nM) and WT PriA (2 nM) were co-incubated with ATP. Unwound products were resolved using TBE-PAGE. **C.** Quantification of the fraction of

DNA replication forks unwound in B. **D.** Quantification of the fraction unwound of DNA replication forks containing ssDNA gaps (5-, 9-, 10-, 11-, 15-bases) between the fork junction and duplexed lagging strand, as depicted in Figure 2D. Normalized to 0 nM PriB. Data points are the mean of at least three independent measurements, with error bars representing the standard deviation.

Supplementary Figure S8: Contacts mediating the PriA CRR^{UP} and CRR^{DOWN} interface stabilization. A. Contacts between H455 and E492 residues with neighboring residues D513, A457, and Q456 stabilize the CRR^{UP} conformation. Distances are shown in Å. **B.** Contacts of helicase motif IV residues R427 and R428 with the ssDNA lagging strand template. **C.** Contacts between H437, H599, and H600 with each other and neighboring residues Q397 and G440 that stabilize the CRR^{DOWN} conformation.

Supplementary Figure S9: Disruption of the PriA CRR position does not impair CRR-Zn^{2+} binding. A. Zn^{2+} standard curve derived from 0 – 25 µM $ZnCl_2$ standards. **B.** Concentration of Zn^{2+} in 2 µM PriA and PriA variant stocks, determined as described in *Materials and Methods*.

Supplementary Figure S10: Destabilization of PriA CRR^{UP} interface impairs PriB binding and PriA DNA helicase activity. A. Quantification of the fraction of PriB-supershifted (PriA/PriB/replication fork complex) to the PriA-shifted (PriA/replication fork) bands in Figure 3C. Normalized to 0 nM PriB. **B.** DNA unwinding by PriA CRR^{UP}-destabilized variants. Fork 4 (1 nM) and WT PriA or $PriA^{CRR}$-destabilized variants (2 nM)

were co-incubated with ATP. Unwound products were resolved using TBE-PAGE. **C.** Quantification of the fraction of DNA replication fork unwound by PriA CRR^{UP}-destabilized in panel B. **D.** Quantification of the PriB-stimulated percentage of unwound DNA, normalized to 0 nM PriB, depicted in Figure 3D. Data points are the mean of at least three independent measurements, with error bars representing the standard deviation.

Supplementary Figure S11: Destabilization of PriA CRR^{DOWN} interface does not impair PriB binding but can reduce DNA helicase activity. A. Quantification of the fraction of PriB-supershifted (PriA/PriB/replication fork complex) to the PriA-shifted (PriA/replication fork) bands in Figure 4B. **B.** DNA unwinding by PriA CRR^{DOWN}-destabilized variants. Fork 4 (1 nM) and WT PriA or $PriA^{CRR}$-destabilized variants (2 nM) were co-incubated with ATP. Unwound products were resolved using TBE-PAGE. **C.** Quantification of the fraction of DNA replication fork unwound by PriA CRR^{DOWN}-destabilized variants in panel B. **D.** Quantification of the PriB-stimulated percentage unwound, normalized to 0 nM PriB, depicted in Figure 4C. Data points are the mean of at least three independent measurements, with error bars representing the standard deviation.

Supplementary Figure S12. PriA and $PriA^{CRR}$ variants do not unwind a DNA replication fork with a ssDNA lagging strand. A. DNA unwinding by PriA CRR^{UP}-destabilized variants on a 3-stranded DNA replication fork. Fork 4 (1 nM) and WT PriA or PriA CRR^{UP}-destabilized variants (0.1, 1, 2, 5 nM) were co-incubated with ATP. Unwound products were resolved in TBE-PAGE. **B.** DNA unwinding by PriA CRR^{DOWN}-destabilized

variants on a three-stranded DNA replication fork. Fork 4 (1 nM) and WT PriA or PriA CRR^{DOWN}-destabilized variants (0.1, 1, 2, 5 nM) were co-incubated with ATP. Unwound products were resolved using TBE-PAGE. **C.** Quantification of the fraction of DNA replication fork unwound by WT PriA and PriA CRR^{UP}-destabilized variants. **D.** Quantification of the fraction of DNA replication fork unwound by WT PriA and PriA CRR^{DOWN}-destabilized variants. Data points are the mean of at least three independent measurements, with error bars representing the standard deviation.

Supplementary Figure S13. Images of selected strains. Representative phase-contrast, nucleoid, and SOS induction images of selected strains as described in the *Materials and Methods*. A 5-micron-sized bar is included in each phase-contrast image for scale.

Supplementary Movie S1. Movie depicting movement between the CRR^{DOWN} and CRR^{UP} positions of the PriA^{CRR}. PriA is colored in grey, except for PriA^{CRR}, which is shown in salmon. The movie in the middle morphs between the CRR^{DOWN} position (left (7)) and the CRR^{UP} position (right (1)).

SUPPLEMENTARY REFERENCES

1. Duckworth,A.T., Ducos,P.L., McMillan,S.D., Satyshur,K.A., Blumenthal,K.H., Deorio,H.R., Larson,J.A., Sandler,S.J., Grant,T. and Keck,J.L. (2023) Replication fork binding triggers structural changes in the PriA helicase that govern DNA replication restart in *E. coli*. *Nat. Commun.*, **14**, 2725.
2. Heller,R.C. and Marians,K.J. (2005) Unwinding of the nascent lagging strand by Rep and PriA enables the direct restart of stalled replication forks. *J. Biol. Chem.*, **280**, 34143–34151.
3. Singer,M., Baker,T.A., Schnitzler,G., Deischel,S.M., Goel,M., Dove,W., Jaacks,K.J., Grossman,A.D., Erickson,J.W. and Gross,C.A. (1989) A Collection of Strains Containing Genetically Linked Alternating Antibiotic Resistance Elements for Genetic Mapping of *Escherichia coli*. *Microbiol Rev*, **53**, 1–24.
4. Sandler,S.J., Marians,K.J., Zavitz,K.H., Coutu,J., Parent,M.A. and Clark,A.J. (1999) *dnaC* mutations suppress defects in DNA replication- and recombination-associated functions in *priB* and *priC* double mutants in *Escherichia coli* K-12. *Mol. Microbiol.*, **34**, 91–101.
5. Sandler,S.J., Samra,H.S. and Clark,A.J. (1996) Differential Suppression of *priA2::kan* Phenotypes in *Escherichia coli* K-12 by Mutations in *priA*, *lexA* and *dnaC*. *Genetics*, **143**, 5-13.
6. Baba,T., Ara,T., Hasegawa,M., Takai,Y., Okumura,Y., Baba,M., Datsenko,K.A., Tomita,M., Wanner,B.L., Mori,H., *et al.* (2006) Construction of *Escherichia coli* K-12 in-frame, single-gene knockout mutants: the Keio collection. *Mol. Syst. Biol*, **2**, MSB4100050.

7. Windgassen,T.A., Leroux,M., Satyshur,K.A., Sandler,S.J. and Keck,J.L. (2018) Structure-specific DNA replication-fork recognition directs helicase and replication restart activities of the PriA helicase. *Proc. Natl. Acad. Sci. U. S. A.*, **115**, E9075–E9084.

8. Zi Tan,Y., Baldwin,P.R., Davis,J.H., Williamson,J.R., Potter,C.S., Carragher,B. and Lyumkis,D. (2017) Addressing preferred specimen orientation in single-particle cryo-EM through tilting. *Nat. Methods*, **14**, 793–796.
